# IGF1R deficiency in vascular smooth muscle cells impairs myogenic autoregulation and cognition in mice

**DOI:** 10.3389/fnagi.2024.1320808

**Published:** 2024-02-15

**Authors:** Lauren R. Miller, Marisa A. Bickel, Stefano Tarantini, Megan E. Runion, Zoe Matacchiera, Michaela L. Vance, Clara Hibbs, Hannah Vaden, Domonkos Nagykaldi, Teryn Martin, Elizabeth C. Bullen, Jessica Pinckard, Tamas Kiss, Eric W. Howard, Andriy Yabluchanskiy, Shannon M. Conley

**Affiliations:** ^1^Department of Cell Biology, University of Oklahoma Health Sciences Center, Oklahoma City, OK, United States; ^2^Vascular Cognitive Impairment and Neurodegeneration Program, Oklahoma Center for Geroscience and Healthy Brain Aging, University of Oklahoma Health Sciences Center, Oklahoma City, OK, United States; ^3^Department of Neurosurgery, University of Oklahoma Health Sciences Center, Oklahoma City, OK, United States; ^4^The Peggy and Charles Stephenson Cancer Center, University of Oklahoma Health Sciences Center, Oklahoma City, OK, United States; ^5^Department of Health Promotion Sciences, College of Public Health, University of Oklahoma Health Sciences Center, Oklahoma City, OK, United States; ^6^Division of Comparative Medicine, Department of Pathology, University of Oklahoma Health Sciences Center, Oklahoma City, OK, United States; ^7^Pediatric Center, Semmelweis University, Budapest, Hungary; ^8^Eötvös Loránd Research Network and Semmelweis University Cerebrovascular and Neurocognitive Disorders Research Group, Budapest, Hungary

**Keywords:** insulin-like growth factor-1, intracerebral hemorrhage, microhemorrhage, brain, aging, cerebrovascular aging, vascular cognitive impairment

## Abstract

**Introduction:**

Cerebrovascular pathologies contribute to cognitive decline during aging, leading to vascular cognitive impairment and dementia (VCID). Levels of circulating insulin-like growth factor 1 (IGF-1), a vasoprotective hormone, decrease during aging. Decreased circulating IGF-1 in animal models leads to the development of VCID-like symptoms, but the cellular mechanisms underlying IGF-1-deficiency associated pathologies in the aged cerebrovasculature remain poorly understood. Here, we test the hypothesis that vascular smooth muscle cells (VSMCs) play an integral part in mediating the vasoprotective effects of IGF-1.

**Methods:**

We used a hypertension-based model of cerebrovascular dysfunction in mice with VSMC-specific IGF-1 receptor (*Igf1r*) deficiency and evaluated the development of cerebrovascular pathologies and cognitive dysfunction.

**Results:**

VSMC-specific *Igf1r* deficiency led to impaired cerebral myogenic autoregulation, independent of blood pressure changes, which was also associated with impaired spatial learning and memory function as measured by radial arm water maze and impaired motor learning measured by rotarod. In contrast, VSMC-specific IGF-1 receptor knockdown did not lead to cerebral microvascular rarefaction.

**Discussion:**

These studies suggest that VSMCs are key targets for IGF-1 in the context of cerebrovascular health, playing a role in vessel stability alongside other cells in the neurovascular unit, and that VSMC dysfunction in aging likely contributes to VCID.

## Introduction

Vascular cognitive impairment and dementia (VCID) encompasses a spectrum of cognitive alterations with underlying vascular pathology, and negatively affects quality of life in affected individuals (Iadecola et al., 2019). In addition to the public health burden from VCID itself, VCID-associated pathologies contribute to other dementias such as Alzheimer’s disease (AD) (Gorelick and Nyenhuis, 2013; Gorelick et al., 2016; Abdelhak et al., 2020). Hypertension and advanced age (65 years) are major risk factors for VCID. VCID is characterized by pathologies including impaired neurovascular coupling (NVC), increased blood-brain-barrier (BBB) permeability, vascular rarefaction, impaired cerebrovascular autoregulation, cerebral microhemorrhages and other manifestations of vascular fragility and dysfunction (Smeda, 1992; Lott et al., 2004; Kang et al., 2009; Poels et al., 2011, 2012). These factors contribute to cognitive impairment, balance and mobility defects, and other adverse consequences. Extensive evidence has demonstrated the role of the somatotropic axis in cerebrovascular aging, in particular, a role for insulin-like growth factor 1 (IGF-1) (Bickel et al., 2023a). IGF-1 is mainly produced by the liver, but also within the brain, and levels decrease with age. Data from humans and animal models indicate that decreased IGF-1 is associated with worsened cerebrovascular disease and signs of VCID (Gong et al., 2012; Muller et al., 2012; Ungvari and Csiszar, 2012; Sonntag et al., 2013; Tarantini et al., 2017c; Ungvari et al., 2017b). Mouse models of circulating IGF-1 deficiency are an experimental model for accelerated cerebrovascular aging, (Toth et al., 2014, 2015a; Tarantini et al., 2016; Fulop et al., 2019b; Miller et al., 2022) and IGF-1 deficiency worsens or accelerates the development of many age-related vascular pathologies including cerebrovascular autoregulation, highlighting its anti-geronic role (Sonntag et al., 2005; Piriz et al., 2011; Shai et al., 2011; von der Thusen et al., 2011; Higashi et al., 2012, 2019; Muller et al., 2012; Trueba-Saiz et al., 2013; Katsimpardi et al., 2014; Toth et al., 2014, 2015a,^2022^; Pardo et al., 2016; Tarantini et al., 2016, 2017b,c; Labandeira-Garcia et al., 2017; Logan et al., 2018; Ascenzi et al., 2019; Farias Quipildor et al., 2019; Prabhu et al., 2019; Norling et al., 2020).

Our goal is to better understand the cellular and molecular mechanisms linking IGF-1 deficiency to the development of age-related VCID. We aim to identify and understand the vascular cell types that play a role in mediating the vasoprotective effects of IGF-1. One potential IGF-1 responsive target is vascular smooth muscle cells (VSMCs), the contractile cells on large- and medium-sized blood vessels that regulate blood pressure, organ perfusion, and vascular integrity in response to environmental stressors such as hypertension (Webb, 2003; Alberts et al., 2015). VSMCs have physiological functions that are essential to maintain the homeostasis of neuronal tissue and support cognition, and contribute to NVC, BBB integrity, and myogenic autoregulation (Carlson et al., 2008; Toth et al., 2013; Hill et al., 2015; Zhao et al., 2015; Shekhar et al., 2017; Liebner et al., 2018; Claassen et al., 2021; Meng et al., 2021). VSMCs and their extracellular matrix (ECM) are essential for arterial resistance to injury, and ECM remodeling is a key function of VSMCs which is regulated, often as part of phenotypic switching. VSMC phenotypic changes are also a part of the vascular response in cerebrovascular diseases such as ischemic and hemorrhagic stroke (Chen et al., 2013; Poittevin et al., 2014; Wu et al., 2016). Growth factors, including IGF-1, are key regulators of VSMC phenotypic switching, for example IGF-1 plays a protective role in atherosclerotic plaque stability through its actions on VSMCs, and we recently demonstrated that IGF-1 induces cellular hypertrophy and altered expression of ECM genes and ECM remodeling genes in cultured primary rat VSMCs (Risinger et al., 2006; von der Thusen et al., 2011; Fulop et al., 2019b; Bickel et al., 2023a,b). A potential role for IGF-1 on brain VSMCs is highlighted by our previous studies showing that mice with circulating IGF-1 deficiency exhibit pathological cerebrovascular arterial wall ECM remodeling in response to hypertension and impaired cerebrovascular autoregulation (Fulop et al., 2019b). This impaired autoregulatory response and ECM remodeling suggests that circulating IGF-1 deficiency leads to maladaptation of VSMCs to hypertension, raising the question of whether VSMCs would exhibit these maladaptive phenotypes if they were unable to receive direct IGF-1 signals. Support for the ability of VSMCs to respond to circulating factors, such as IGF-1, comes from our parabiosis studies which show that exposure of young mice to aged blood induces a pro-geronic shift in VSMC gene expression (Kiss et al., 2022). However, IGF-1 receptors are expressed in many cell types in the brain, including endothelial cells and astrocytes in addition to VSMCs (Tarantini et al., 2021a,b). This makes it necessary to directly interrogate the VSMC-specific role of IGF-1 to clearly understand the complex interplay by which IGF-1 deficiency contributes to vascular aging.

Here, we probed the role of IGF-1 in VSMCs in the development of VCID associated phenotypes using an adult-onset VSMC-specific Igf1 receptor knockout model. We hypothesized that decreased IGF-1 signaling in VSMCs would lead to impaired myogenic autoregulation in arteries and arterioles, subsequent microvascular damage, and downstream cognitive deficits. We found that VSMC-specific Igf1r deficiency impaired myogenic autoregulatory responses and contributed to impaired spatial and motor learning, modeling pathologies associated with VCID. Our findings demonstrate a critical role for VSMCs in IGF-1 deficiency-related cerebrovascular disease and VCID.

## Materials and methods

### Animal model

Animal experiments were approved by the Institutional Animal Care and Use Committee at the University of Oklahoma Health Sciences Center (Oklahoma City, OK, USA). VSMC-specific IGF-1 receptor (Igf1r) knockdown mice were generated by crossing strains from Jackson Laboratories (Bar Harbor, ME, USA). The mural cell-specific *Myh11-Cre*^*ERT*2^ (B6.FVB-Tg(Myh11-icre/ERT2)1Soff/J, Strain #:019079) was crossed with the IGF-1 receptor flox line (*Igf1r*^*f/f*^, B6;129-Igf1rtm2Arge/J, Strain #:012251) to generate an inducible mural cell-specific *Igf1r* knockdown line. For some experiments, we additionally crossed these animals onto the tdTomato lineage tracer line [*ROSA*^*f/ftdT*^ B6.Cg-Gt(ROSA)26Sortm9(CAG-tdTomato)Hze/J, Strain #:007909] in which excision of a floxed STOP cassette results in permanent expression of the tdTomato reporter in all cells expressing Cre. This resulted in our triple-crossed line with inducible mural cell-specific *Igf1r* knockdown and Tomato reporter (*Myh11-Cre*^*ERT*2^ *ROSA*^*f/ftdT*^ *Igf1r*^*f/f*^). Control animals were *Myh11-Cre*^*ERT*2^ *ROSA*^*f/ftdT*^ *Igf1r*^+/+^ mice. Genotyping information is in Supplementary Table 1. Knockdown was induced at 4 months of age (tamoxifen, 75 mg/kg/day, 5 days) Male mice were used as the *Myh11-Cre*^*ERT*2^ is on the Y-chromosome. Mice were housed in the OUHSC Rodent Barrier Facility under specific pathogen-free conditions. Prior to experiments, animals were transferred to conventional rodent housing facilities at OUHSC. Animals were exposed to a 12 h light/12 h dark cycle, with access to standard rodent chow (Purina Mills, Richmond, IN, USA) and water *ad libitum*. All experiments and analysis were performed by a lab member blinded to treatment group and genotype.

### Hypertension induction and blood pressure measurements

Hypertension was induced at 10−12-months of age. *Myh11-Cre*^*ERT*2^ *ROSA*^*f/ftdT*^ *Igf1r*^*f/f*^ and *Myh11-Cre*^*ERT*2^ *ROSA*^*f/ftdT*^ *Igf1r*^+/+^ mice were randomly assigned to either “hypertensive” or “normotensive” experimental groups. Mice were implanted with subcutaneous osmotic mini-pumps (Alzet Model 2006, 0.15 μL h^–1^, Durect Co, Cupertino, CA, USA) filled with either sterile saline or angiotensin II in saline (Millipore Sigma, St. Louis, MO, USA) delivered at a rate of 1 μg min^–1^kg^–1^ for the duration of the experiment using established methods (Toth et al., 2013, 2014; Fulop et al., 2019b). Under aseptic conditions, an incision was made in the intrascapular region in isoflurane-anesthetized mice. The minipump was inserted into the subcutaneous space and the incision was closed with surgical sutures.(Tarantini et al., 2016; Miller et al., 2022) Mice were given subcutaneous extended-release buprenorphine (ZooPharm, Fort Collins, CO, USA) for post-operative pain. Blood pressure was measured ∼2 weeks post-pump implantation via the tail cuff method (CODA Non-Invasive Blood Pressure System, Kent Scientific Co., Torrington, CT, USA) (Toth et al., 2015b). Hypertensive mice were divided into two hypertension paradigms. For autoregulation measurements, and related experiments, mice were subjected to hypertension for 4 weeks before measurements and tissue collection (designated group 1). In a different subset of mice, behavior experiments began 4 weeks post-hypertension induction and mice were collected at 7 weeks post-pump implantation (designated Group 2) for related experiments.

### Standardized neurological examination and removal criteria

Starting 3 days post-pump implantation, standardized neurological examinations were performed daily for 1 week. After week one, examinations were performed biweekly until the conclusion of the experiment. Scoring used an 18-point scale that assesses body proprioception, response to vibrissae stimulation, symmetry in limb movement, forelimb outstretching, climbing ability, and spontaneous activity as described (Nyúl-Tóth et al., 2020; Miller et al., 2022). A decline in neurological score below 15 indicates hemorrhagic strokes and is a humane endpoint.

### Protein isolation and western blots

Aortas harvested after cardiac perfusion were flash frozen in in liquid nitrogen. For protein extraction, aortas were minced on ice and placed in SMAD lysis buffer [10 mM Tris-Hcl pH 7.6, 1.0% Triton X-100, 100 mM NaCl, 2.0 mM EDTA, 10% v/v glycerol containing 50 mM NaF, 20 mM Na_4_P_2_O_7_, 2.0 mM Na_3_VO_4_ and 1x protease inhibitor cocktail (Roche, Indianapolis, IN)], sonicated, and then incubated on a rocking platform at 4°C for 30 min. Insoluble material was removed by centrifugation at 15000 × g for 10 min at 4°C, and protein was quantified using Bradford reagent (Bio-Rad, Temecula, CA). SDS-PAGE and western blots were performed according to standard protocols (Conley et al., 2019). Blots were incubated overnight in primary antibodies (Table 1) and in secondary antibodies (Table 1) for 1 h. Blots were imaged using a Licor Odyssey Fc imager (Licor). Densitometry on unsaturated bands was performed using Image Studio (v.5.2, Licor).

**TABLE 1:** Antibodies.

**Antigen**	**Species**	**Use and dilution**	**Source**	**RRID**	**References**
ASMA	Ms-MC	IF (1:500), WB (1:1000)	Millipore Sigma, cat# 113200 clone 1A4	RRID:AB_564184	Jackson-Weaver et al., 2020
Endomucin	Rat-MC	IF (1:200)	Millipore Sigma, cat# MAB2624, clone V.5C7	RRID:AB_10807039
Igf1R	Rbt-MC	IF (1:200), WB (1:500)	Abcam, cat# ab182408	N/A	Liu and Khalil, 2018
B -Actin-HRP	Ms-MC	WB (1:10000)	Sigma, cat# A3854	RRID:AB_262011	
Pdgfr-beta	Rbt-MC	IF (1:500)	Abcam, cat# ab32570,	RRID:AB_777165	
CD31	Rat-MC	IF (1:200)	BD Sciences cat# 550274	RRID:AB_393571	
Goat anti-Mouse 680	Gt-PC	WB (1:10,000)	Licor Biosciences Cat# 926-68022	RRID:AB_10715072	
Goat anti-Rabbit 800	Gt-PC	WB (1:10,000)	Licor Biosciences Cat# 926-68071	RRID:AB_10956166	
Alexa fluor 647-anti-mouse	Gt-PC	IF (1:200)	Thermo Fisher Scientific/Invitrogen, cat# A21242;	RRID:AB_2535811	
Alexa fluor 488-anti-rabbit	Gt-PC	IF (1:200)	Thermo Fisher Scientific/Invitrogen, cat# A11034	RRID:AB_2535811	
Alexa fluor 647-anti-rabbit	Gt-PC	IF (1:200)	Thermo Fisher Scientific/Invitrogen, cat# A31573	RRID:AB_2535811	

### Enzyme-linked immunosorbent assay (ELISA)

For serum IGF-1 measurements, blood was collected from the submandibular vein of a subset of normotensive and hypertensive (4 weeks, Group 1) mice from each group with a 25-gauge needle. Mice were fed *ad libitum* throughout the study, including prior to blood collection. Collected blood was allowed to coagulate for 20 min at room temperature. After coagulation, blood samples were subjected to centrifugation at 2500 × g for 20 min at 4°C. Serum was collected and stored at −80°C until use. For cortical IGF-1 measurements, cerebral cortex was collected from a different perfused mice from each group (7 weeks, Group 2) and flash frozen in liquid nitrogen. Frozen cortexes were stored at −80°C until use. For protein extraction, cortexes were placed in RIPA lysis buffer [50 mM Tris pH 7.4, 1.0%, 150 mM NaCl, 1.0 mM EDTA, NP40, 1.0%, sodium deoxycholate, 0.5%, SDS, 0.1%, 1 mM PMSF, 1 mM protease inhibitor cocktail (Millipore Sigma, St. Louis, MO, USA)], sonicated, and then incubated on a rocking platform at 4°C for 30 min. Insoluble material was removed by centrifugation at maximum speed (20,000 × g) for 25 min at 4°C, and protein was quantified using Bradford reagent (Bio-Rad, Temecula, CA). Serum and cortex IGF-1 levels were measured by ELISA (R&D Systems, Minneapolis, MN, USA) as previously described (Toth et al., 2014). On each plate an IGF-1 positive control sample was included. IGF-1 levels are reported as ng mL^–1^ for serum levels and pg/mg protein for cortex samples. Protein levels in cortex samples were assayed using Bradford reagent according to manufacturer’s instructions.

### Autoregulation and neurovascular coupling measurements

Myogenic autoregulation measurements were acquired using a modified version of our previously reported method for measuring autoregulation and NVC (Toth et al., 2015a; Tarantini et al., 2018; Fulop et al., 2019b; Miller et al., 2022). Measurements for autoregulation were conducted on 11−13-month-old mice 27−29 days post-minipump implantation. Intraperitoneal α-chloralose [50 mg/kg]/urethane [750 mg/kg] was administered to mice 30 min before the start of surgical procedures to maintain long-term anesthesia. Mice were additionally anesthetized with 1−2% isoflurane to ensure depth of anesthesia during surgical preparations. A femoral artery cannula was placed to measure and control blood pressure. Mice were endotracheally intubated and mechanically ventilated (MousVent G500; Kent Scientific Co., Torrington, CT, USA) with rectal temperature maintained at 37°C using a heating pad (Kent Scientific Co.). After intubation, the mice were placed in a stereotaxic frame (Leica Microsystems, Buffalo Grove, IL). A thinned-skull cranial window was prepared by resecting the scalp and periosteum and then thinning the skull with a sterile scalpel blade. To allow for appropriate optics, nitrocellulose lacquer was applied to the surface of the skull. The mice were placed under a laser speckle imager (Pericam PSI System, Perimed, Järfälla, Sweden) adjusted to 10 cm above the mouse. Isoflurane was removed, and baseline cerebral blood flow (CBF) was recorded using the laser speckle imager. Mice then underwent laser speckle imaging as blood pressure was increased by infusing phenylephrine (1−2 μg/kg per minute) into the femoral artery cannula via a peristaltic pump. Blood pressure started at 80 mmHg (near the natural starting pressure) and increased every ∼5 min as follows: 80, 100, 120, 140, 150, 160, 170, 180, 190 mmHg, and maximum pressure of the peristaltic pump (192−200 mmHg). After the CBF stabilized, the average relative perfusion at each step was calculated and normalized to baseline. Graphed values were averages over an ∼30 s time window taken at each step after blood pressure had stabilized at each pressure in a region of interest (ROI) defined at the beginning of each experiment and corresponding to the majority of the exposed brain surface (marked on relevant figures), calculated from within the PIMSoft software that runs the Perimed PSI system. At the end of the experiment, mice were transcardially perfused with 1x phosphate buffered saline (1x PBS, 137 mM NaCl, 2.7 mM KCl 10 mM Na_2_HPO_4_, 1.8 mM KH_2_PO_4_, pH 7.2−7.4) for 10 min prior to tissue harvesting.

Neurovascular coupling (NVC) responses were recorded in a different subset of normotensive 10−12-month-old mice (designated Group 3) as previously reported (Tarantini et al., 2017a,^2018^; Yabluchanskiy et al., 2020; Miller et al., 2022). Mice were anesthetized with 4% isoflurane prior to surgery. Surgery and laser speckle imager positioning was performed as for autoregulation. Isoflurane was reduced to 1%, the whiskers on alternating sides of the face were stimulated in six 30 s intervals and changes in CBF to the contralateral whisker barrel cortex were recorded. Each stimulus was preceded by 30 s of imaging with no stimulation. ROIs were placed over the barrel cortex on each side of the brain at the beginning of each experiment and a standard sized ROI was used across all mice in the cohort. Graphed data reflect values averaged over ∼5-10 s corresponding to the maximum plateau reached during whisker stimulation from the ROI on the stimulated side of the brain. These values were normalized to pre-stimulation baseline levels from the same ROI. Data are displayed as CBF increase from baseline measurements.

The difference images in our study were generated using the PeriCam PSI System, based on Laser Speckle Contrast Analysis (LASCA) technology. Initially, we captured baseline cerebral blood flow (CBF) images before any whisker stimulation to serve as a reference for comparison. Subsequent images during and after whisker stimulation were then captured, reflecting changes in CBF in response to the stimulus. The difference images were calculated by subtracting the baseline speckle contrast image from the stimulus-response speckle contrast images, highlighting changes in CBF due to the stimulation. Red pixels reflect regions of increased flow from baseline and blue pixels reflect regions of decreased flow from baseline. All images were normalized against baseline measurements to account for any inter-session variability, and instrument calibration was performed to ensure that measurements were consistent and reproducible.

#### Analysis of spatial and motor learning and memory

Behavioral assays began 4 weeks after minipump implantation in a second cohort of mice (designated Group 2) that were not used for autoregulation or NVC measurements. Prior to behavioral assays, mice were housed for at least 1 week in the behavioral testing room for acclimation. The radial arm water maze assay (RAWM) was performed as described (Ungvari et al., 2017a; Fulop et al., 2019a; Tarantini et al., 2019). The eight maze arms (7.5 cm wide) radiate from a central area. One arm contained a submerged escape platform. The water in the maze contained white food dye to obscure the escape platform. The water temperature was 20−24°C (tested daily). A privacy curtain surrounded the maze to minimize distractions. Intramaze cues (black and white shapes) were placed at the end of each arm. Video tracking with Ethovision (v.14 for acquisition and v.16 for analysis) from Noldus Information Technology Inc. (Leesburg, VA, USA) was used to record data. Each mouse started in a pseudo-randomly selected arm that did not contain the escape platform and swam for up to 1 min to find the platform. The learning phase lasted 3 days and consisted of acquiring two sets of four trials for each mouse. At the end of the first trial on day one, mice were placed on the hidden platform to learn its position. Combined error rate was calculated as:


[CER=(#ofincorrectarmentries)+(secondsspentincenter÷15)

Learning was assessed by comparing trial sets across 3 days. After the learning phase, mice returned to their home cages for 7 days. On day 10, long-term memory was assessed with a probe set of four trials with the platform located in the same arm as before.

To assess motor coordination and learning, an automated four-lane rotarod (Columbus Instruments, Columbus, OH, USA) was used as described (Tarantini et al., 2015; Fulop et al., 2019a). Three consecutive measurements per day per mouse were performed on four consecutive days. The apparatus accelerated from 4-40 rpm in 300 s, with the speed increasing every 10 s. The rpm and the time of each fall was recorded by an infrared laser beam (Deacon, 2013; MacLaren et al., 2014). Mice rested for 5 min between runs.

## Y-Maze

The Y-maze assay for spatial memory was performed as previously described on the same cohort of mice as other behavioral assays (Tarantini et al., 2015). The maze consisted of three connected opaque plastic arms that are 7.5 cm wide with a removeable divider that could be used to block the entrance to one arm. A privacy curtain was used around the maze to prevent distractions. Extramaze cues were used as described for the RAWM assay. The maze was cleaned with 70% ethanol between trials. The same video tracking equipment and software was used as described for the RAWM (see above). Mice were always placed in the same arm to start (designated as the “home arm”). During the acclimation phase, the mice were allowed to spend 5 min in the maze with only two arms available to explore (the home arm and the adjacent “familiar arm”). The mice were then returned to their home cages for 4 h. After 4 h, the mice were re-introduced to the maze for a probe phase with the third, “novel arm” available to them in addition to the other two arms. The mice were allowed to explore all three arms of the maze for 2 min. This test relies on the natural exploratory behavior of mice and assesses their ability to recognize that the “novel arm” is a new space. The percentage of time spent in each arm and the total number of entries into each arm serve as a measure of exploratory behavior. The novelty index measures the percent time that a mouse spent in the novel arm compared to the familiar and home arms and is calculated as follows:


$$N\,o\,v\,e\,l\,t\,y\,I\,n\,d\,e\,x\%\;=\frac{c\,u\,m\,u\,l\,a\,t\,i\,v\,e\,d\,u\,r\,a\,t\,i\,o\,n\,i\,n\,n\,o\,v\,e\,l\,a\,r\,m}{c\,u\,m\,u\,l\,a\,t\,i\,v\,e\,d\,u\,r\,a\,t\,i\,o\,n\,i\,n\,n\,o\,v\,e\,l+f\,a\,m\,i\,l\,i\,a\,r\,a\,r\,m\,s}*100$$

A spontaneous alternation is counted when on three consecutive arm entries, a mouse enters a new arm each time (Miedel et al., 2017). Percent spontaneous alternation was calculated as follows:


S⁢p⁢o⁢n⁢t⁢a⁢n⁢e⁢o⁢u⁢s⁢A⁢l⁢t⁢e⁢r⁢n⁢a⁢t⁢i⁢o⁢n% =#⁢o⁢f⁢s⁢p⁢o⁢n⁢t⁢a⁢n⁢e⁢o⁢u⁢s⁢a⁢l⁢t⁢e⁢r⁢n⁢a⁢t⁢i⁢o⁢n⁢s⁢i⁢n⁢ 3⁢a⁢r⁢m⁢st⁢o⁢t⁢a⁢l⁢#⁢o⁢f⁢a⁢r⁢m⁢e⁢n⁢t⁢r⁢i⁢e⁢s-2*100

### Grip strength

Muscle strength of mouse forelimbs was measured using a grip strength meter (Chatillon Ametek Force Measurement, Brooklyn, New York, USA) on the same mice utilized in other behavioral assays. Each mouse had three measurements in three trial blocks (nine measurements per mouse). Measurements were performed by the same investigator throughout. Grip strength values for each mouse were averaged.

#### Immunofluorescence

**FIGURE 1:**
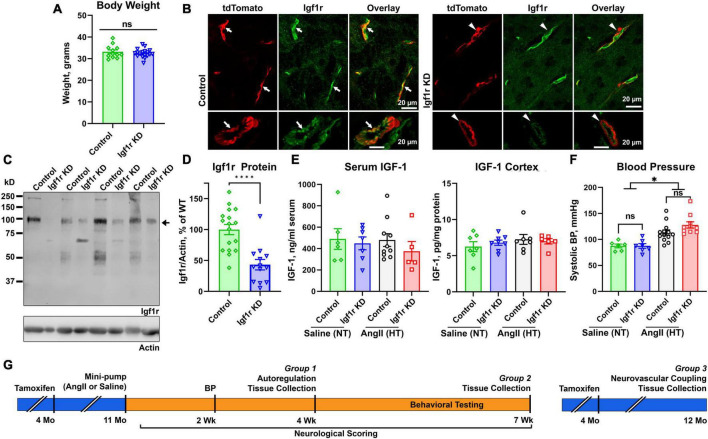
Effective knockdown in VSMC-specific Igf1r mouse model. Igf1r KD and control mice were injected with tamoxifen to induce knockdown at 4 months of age. **(A)** Body weights at 10 months of age were compared between normotensive control and Igf1r KD animals. **(B)** Frozen brain sections from ∼1 year old animals were immunolabeled for Igf1r (green) and imaged with native tdTomato fluorescence. **(C)** Western blot was performed on aortic lysates harvested at ∼1 year of age from normotensive mice. The bottom band at ∼100kDa represents the mature Igf1r, while the larger bands represent the Igf1r proprotein (Khatib et al., 2001). Igf1r densitometry signal was normalized to β-actin levels and plotted in **(D,E)**. ELISA was performed to evaluate IGF-1 levels in serum (4 weeks post-pump implantation, Group 1 animals) and brain cortex (Group 2 animals, 7 weeks post-pump implantation). **(F)** The tail cuff blood pressure measurement method was used to measure blood pressure ∼2 weeks post-pump implantation. **(G)** Shown is a timeline of experiments, mice were injected with tamoxifen to induce knockdown of VSMC Igf1r at 4 months of age. For Groups 1 and 2, at 11 months of age, mice were randomly assigned to a treatment group and implanted with either saline or AngII minipumps. Neurological scoring began day 3 post-surgery and continued until the end of the 4 week period. Blood pressure was measured after ∼2 weeks post-surgery to ensure hypertension. At 4 weeks post-surgery, mice either underwent terminal autoregulation measurements followed by tissue collection (Group 1) or began a series of behavioral cognitive assays which were completed at 7 weeks post-pump implantation and followed by tissue collection (Group 2). Group 3 consisted of only normotensive mice who were injected with tamoxifen as in other groups but did not receive minipumps/hypertension. Group 3 mice underwent terminal assessment of neurovascular coupling measurements followed by tissue collection at 1 year of age. **(A, D)** (*n* = 13–17 mice/group; ****P < 0.0001) two-tailed unpaired *t*-test. **(E)** (*n* = 5–7 mice/group) **(F)** (*n* = 6–13 mice/group; *P < 0.05) analyzed by two-way ANOVA with Tukey’s *post-hoc* comparison. Plotted are mean ± SEM with data points representing individual animals. Scale bars: 20 μm.

Animals were perfused with 1x PBS 4 weeks or 7 weeks after minipump implantation (i.e., after autoregulation experiments or behavioral experiments were completed). Brains from both cohorts of mice were harvested, fixed (48 h in 4% paraformaldehyde PFA), cryoprotected in 10−30% sucrose, embedded (OCT, ThermoFisher, Waltham, MA, USA), and cryosectioned. 70 μm coronal sections were taken −2.3 mm (+/−0.2) from bregma. Immunofluorescence was performed as described (Miller et al., 2022). Slides were pre-treated with 3% hydrogen peroxide (10 min), washed in 1x PBS, treated with 1% sodium borohydride (5 min), washed with water and 1x PBS. Slides blocked (5% BSA, 1% fish gelatin, 0.2% donkey serum, and 0.05% Triton in 1x PBS) for 2 h at room temperature. Slides were incubated in primary antibody (Table 1) for 48 h at 4°C, in secondary antibodies for 2 h at room temperature, and were mounted with Prolong Diamond with DAPI (ThermoFisher). Fluorescent imaging was performed using a Super-resolution Nikon CSU-W1/SoRa spinning disk confocal microscope at 40x, or an Olympus BX-62 (Olympus USA, Center Valley, PA) microscope at 40x equipped with a spinning disc confocal unit and a Hamamatsu C-4742 camera. Images in Figure 1 and Supplementary Figure 1 are single planes from a confocal stack. For rarefaction studies, slides were imaged on a Leica M205-MFC THUNDER microscope at 16x, and image tiling was performed using the Leica LasX software (Figures 5, 6, Supplementary Figures 4, 5). From the tiled image, 2500 × 2500-pixel regions were extracted in the hippocampus, cortex, and thalamus.

### Analysis of vascular density

Immunofluorescent images with endothelial cell labeling were analyzed using AngioTool and/or MATLAB software. From the overall stitched image of the brain slice, 2500 × 2500-pixel images were extracted in the hippocampus, cortex, and thalamus. AngioTool software (v0.6) was used to quantify vascular network parameters (average vessel length, vessel percent area, total vessel length, lacunarity, etc.) (Zudaire et al., 2011). Analysis was performed by a lab member blinded to treatment group and genotype. AngioTool results were validated using a Matlab (version R2022b) script. The script processed .jpeg images into a simple binary and an enhanced contrast binary image after background subtraction. Background subtraction was performed using a rolling ball method. The subtracted image was converted into a simple binary using the imbinarize function and Otsu’s thresholding which minimizes the intraclass variance of the thresholded black and white pixels. To produce the enhanced contrast binary images, the script used the average intensity of the original image to set the ClipLimit value of the adapthisteq function. This function uses the contrast-limited adaptive histogram equalization (CLAHE) algorithm. The average intensity of the image was found by applying the function mean2. Percentages of vascular density from both the simple binary and the enhanced contrast binary images were exported along with a montage image.

To assess changes in microvasculature without the presence of large vessels, images captured with both CD31/endomucin labeling and tdTomato native fluorescence were used. Using ImageJ/FIJI, images underwent background subtraction using a rolling ball method followed by contrast enhancement using the CLAHE method. Binary images were created using Otsu’s thresholding for each channel. ROIs were created around only VSMC covered vessels using but not pericyte covered vessels using the binary tdTomato image. These ROIs were applied to the CD31/endomucin binary image and using the invert function, a new ROI was created corresponding to the entire image except the VSMC covered vessels. The vessel percent area in this new ROI lacking VSMC-covered vessels was calculated using the analyze function in ImageJ/FIJI. Because the tdTomato reporter labels VSMCs and a subset of pericytes (found on smaller vessels), determination of which vessels were VSMC-covered (and thus exclude from this analysis) was made based on the morphology of the tdTomato cells in the original tdTomato images. Pericytes exhibited a characteristic stringy or mesh-like morphology with pronounced visible nuclei, while VSMCs exhibited more uniform vessel coverage, lacked visible pronounced nuclei, and covered larger vessels. All steps in image analysis were performed by lab members blinded to treatment group.

### Analysis of gene expression

Hippocampus and cerebral cortex were collected from transcardially perfused normotensive and hypertensive mice (7 weeks post pump-implantation, Group 2) and flash frozen in liquid nitrogen. Total RNA was isolated using RNeasy Mini QIAcube Kit according to the manufacturer’s instructions (cat. no. 74116, Qiagen, Germantown, MD, USA). RNA concentration was assessed using a DeNovix DS-11 spectrophotometer. Total RNA was reverse transcribed into cDNA using the High-Capacity RNA-to-cDNA Kit according to the manufacturer’s instructions (cat. no. 4387406, ThermoFisher) using a Bio-Rad T100 thermocycler (Bio-Rad, Temecula, CA, USA). Two TaqMan Gene Expression Custom Array Cards were designed to test 96 qPCR targets each associated with vascular function (Supplementary Table 2) (cat. no. 4342259, ThermoFisher). Each port of a microfluidic card was loaded with 100 μl of reaction mixture including 50 μl of TaqMan Universal Master Mix II with UNG (cat. no. 4440038, ThermoFisher), 40 μl of nuclease-free water, and 10 μl of cDNA. Each card included a sample from each group. Cards were centrifuged prior to sealing and removing ports. qPCR was performed using the QuantStudio 12K Flex Real-Time PCR System.

Normalization of qPCR data was performed using the efficiency−corrected ΔΔCq method. The analysis was performed in R environment (R v.4.3.1). Relative quantities of the reference genes *Hprt*, *Ywhaz*, *B2m*, and *Actb* were determined, and a normalization factor was calculated based on the geometric mean for internal normalization. Ct values missing due to low amount of original sample were replaced with 39, the highest possible Ct value.

PANTHER (v18.0) was utilized for gene ontology (GO) analysis. For each gene or gene set, the PANTHER overrepresentation test tool was used to compare individual genes or gene lists to a reference list obtained from the GO Ontology database ( doi: 10.5281/zenodo.7942786 Released 2023-01-05).^1^ For each gene or gene list, the tool determined biological processes in which gene(s) were overrepresented. Significance was calculated via a Fisher’s Exact test. For individual gene comparisons to the reference list, pathways in which the genes were enriched were determined by the calculated raw *p*-value (*P* < 0.05). For gene set comparisons to the reference list, pathways in which the gene set was enriched were determined by the *p*-value corrected for False Discovery Rate (FDR) (*P* < 0.05).

## Statistical analysis

Statistical analyses were performed using GraphPad Prism v.9.2. Unpaired *t*-tests were used when two samples were compared, and two-way ANOVA with Tukey’s *post-hoc* test was used when multiple variables were included (i.e., genotype and hypertension). Three-way ANOVA was used to analyze the learning phase for RAWM data and rotarod data (variables: time, genotype, treatment), as well as autoregulation data [variables: mean arterial pressure (MAP), genotype, treatment (hypertensive vs. normotensive)]. Linear regression was used to assess autoregulation data and slopes were compared using an ANCOVA (analysis of covariance)-related method adapted from (Zar, 2010). Significance was set at *P* < 0.05. Graphs plot mean ± SEM, with individual data points shown representing individual animals.

## Results

### Generating a model of VSMC-specific Igf1r deficiency

We generated an inducible VSMC-specific IGF-1 receptor (Igf1r) knockdown (*Myh11-Cre*^*ERT*2^ *Igf1r*^*f/f*^, abbreviated Igf1r KD) either with or without the ROSA26 lox-stop-lox tdTomato lineage tracing reporter (*ROSA26*^*f/ftdT*^). Recombination was induced via tamoxifen at 4 months of age (Supplementary Figure 1A) to prevent effects arising due to the role of IGF-1 in development. As part of initial characterization of this model, we assessed body weights, circulating IGF-1 levels, and IGF-1 levels in the brain cortex at 1 year of age, and as expected, there were no differences between Igf1r KD (*Myh11-Cre*^*ERT*2^ *Igf1r*^*f/f*^) and control mice (*Myh11-Cre*^*ERT*2^ *Igf1r*^+/+^) (Figures 1A, E). In the brain, the *Myh11* Cre driver is expressed in VSMCs and a subset of pericytes [which express contractile markers at varying levels (Attwell et al., 2016; Kisler et al., 2017; Sweeney et al., 2018)] as shown in Supplementary Figure 1B (yellow arrows show VSMCs expressing tdTomato, blue arrows show pericytes expressing tdTomato, and green arrows show pericytes not expressing tdTomato). Igf1r is expressed in both brain VSMCs and endothelial cells; Figure 1B shows brain sections from VSMC-tdTomato animals (Igf1r KD or control) labeled for Igf1r. Arrows show co-localization of tdTomato (red) and Igf1r (green) in control sections while arrowheads highlight absence of co-localization in Igf1r KD sections. To quantitatively assess knockdown, we evaluated Igf1r protein levels in the VSMC-rich aorta. Knockdown efficiency varied (and aorta samples also contain endothelial cells), but we observed ∼60% knockdown of Igf1r in Igf1r KD aorta extracts vs. WT (arrow, Figures 1C, D). The ∼100 kDa band corresponds to the Igf1r β-subunit; the larger band is Igf1r proprotein (Khatib et al., 2001).

**FIGURE 2:**
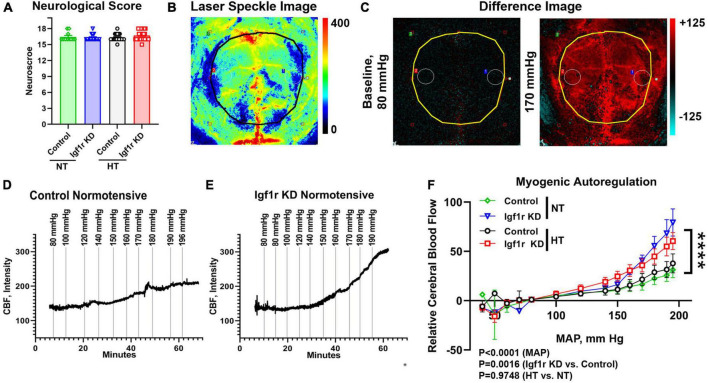
VSMC-specific Igf1r KD impairs myogenic autoregulatory responses. To assess myogenic autoregulatory capacity in the VSMC-specific Igf1r KD model, we used laser speckle contrast imaging to measure changes in blood flow at increasing blood pressures on the cortical surface in mice with thin-skull cranial windows. **(A)** Shown is neurological score at 4 weeks post-pump implantation (prior to autoregulation measurements). **(B)** Example pseudocolor laser speckle image and difference images **(C)** of the visualized cortical surface measured by the laser speckle doppler device. In difference images, red indicates areas of blood flow that are elevated compared to baseline while blue indicates areas of low blood flow reduced compared to baseline. Black/yellow outlines in panel **(B,C)** highlight the region used for analysis. Images in panel **(B,C)** come from the animal whose trace is depicted in **(E)**. Example images [including the ones shown in panel **(B,C)**] from each pressure step corresponding to the traces in **(D,E)** are shown in Supplementary Figure 2. **(D,E)** Example raw traces showing the changes in cerebral blood flow (CBF) over time with increased mean arterial blood pressure (MAP) in control normotensive and Igf1r KD normotensive mice. **(F)** Relative CBF as a function of MAP was plotted for control and Igf1r KD mice that were either hypertensive or normotensive for 4 weeks prior to measurements (Group 1). **(F)** (*n* = 10–11 mice/group; ****P < 0.0001) Igf1r KD vs. control slopes measured by linear regression. Differences between groups were also assessed using three-way ANOVA (variables MAP, normotensive [NT] vs. hypertensive [HT] and genotype), *p* values shown under **(F)**. **(A)** (*n* = 11–28 mice/group) Plotted are mean ± SEM with data points representing individual animals.

Hypertension exacerbates many cerebrovascular consequences of circulating IGF-1 deficiency. Therefore, in addition to comparing outcomes in Igf1r KD vs. control animals, we also assessed the effects of hypertension in many experiments. We used a well-validated model to induce mild elevations in blood pressure via infusion of angiotensin II (AngII) (Toth et al., 2013, 2014; Fulop et al., 2019b). At ∼10−12 months of age, control and Igf1r KD mice were implanted with subcutaneous osmotic minipumps releasing either AngII or saline (Figures 1F, G). Experimental mice were divided into three groups. Group 1 animals underwent assessment of myogenic autoregulation at 4 weeks post-pump implantation followed by tissue collection. Group 2 animals underwent behavioral analyses beginning 4 weeks after pump implantation followed by tissue collection at the end of behavioral experiments (7 weeks, Group 2 Figure 1G). Mini-pumps were implanted at ∼1 year of age since circulating IGF-1 knockdown mice at this age mimic phenotypes seen in aged mice (∼2 years of age). AngII infusion led to ∼35% increase in blood pressure at ∼2 weeks after pump implantation (Figure 1F) vs. saline infusion (hereafter labeled hypertensive: HT and normotensive: NT) with no significant difference between control and Igf1r KD. Standard neurological examination was performed throughout the hypertensive period since severe hypertension can induce hemorrhagic stroke (indicated by a drop in neurological score below 15). We did not observe any significant differences in neurological score between groups, (Figure 2A shows neurological score at 4 weeks post pump-implantation), suggesting the hypertension paradigm was well-tolerated.

### Igf1r-deficiency in VSMCs causes impaired myogenic autoregulation and neurovascular coupling

One of the critical functions of VSMCs is maintaining the cerebrovascular autoregulatory response, in which arteries and arterioles constrict in response to increased systemic blood pressure to maintain consistent blood flow in capillaries (Carlson et al., 2008; Claassen et al., 2021). This response helps protect the microvasculature from the deleterious effects of increased or variable systemic pressure. To assess whether VSMC-specific *Igf1r* knockdown affected cerebral myogenic autoregulation, we examined cerebral blood flow (CBF) at steadily increasing blood pressures in normotensive and hypertensive Igf1r KD and control mice. At ∼4 weeks after mini-pump implantation, laser speckle imaging (Figures 2B, C) was used to measure changes in CBF on the surface of the brain in response to stepwise increases in blood pressure (induced by infusion of phenylephrine in saline). Relative change in CBF was assessed by subtracting baseline values averaged over the brain surface (black/yellow outlines in Figures 2B, C and Supplementary Figure 2 indicate area analyzed) from values collected at increasing pressures (visually depicted as difference images, Figure 2C). Representative raw traces plotting relative CBF over time (with induced pressure changes marked with vertical lines) are shown in Figures 2D–E, and representative images corresponding to each pressure step from these two traces are shown in Supplementary Figure 2. The slope of the pressure-CBF curve was significantly higher in Igf1r-knockdown animals compared to controls, indicating impaired myogenic autoregulatory responses (Figure 2F). Consistent with this, the autoregulatory range was larger in control mice [80−170 mmHg] than in Igf1r KD mice [80−140 mmHg]. This is the pressure range (assessed by repeated measures ANOVA) that shows no statistically significant increase in blood flow. There were significant differences in CBF between genotypes (Igf1r KD vs. control, *P* = 0.0016 by 3-way ANOVA) and across MAP during the experiment (*P* < 0.0001), but not between normotensive and hypertensive animals. These data suggest that decreased IGF-1 signaling in VSMCs leads to impaired autoregulatory function independent of pre-existing hypertension.

**FIGURE 3:**
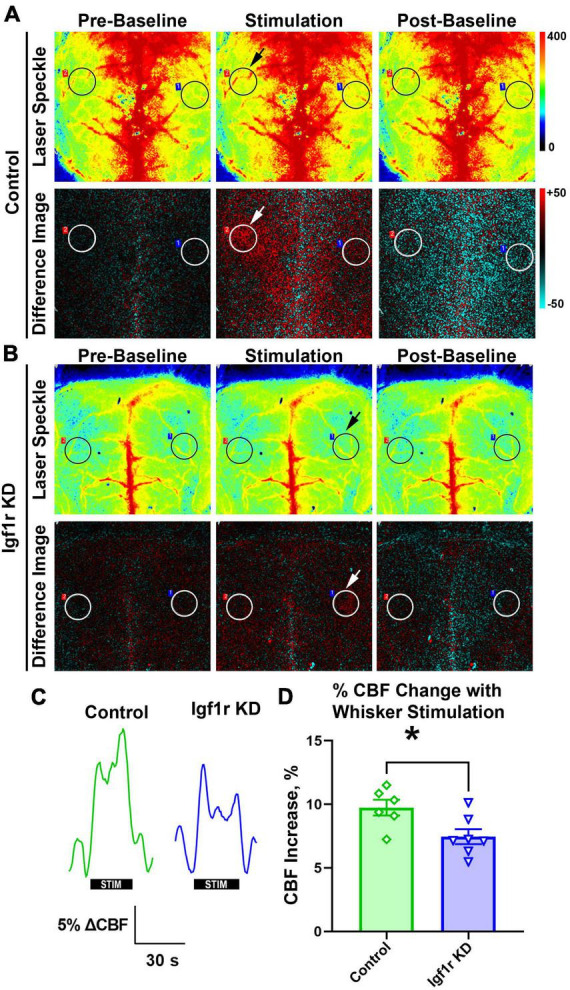
Igf1r KD impairs neurovascular coupling responses. Normotensive Igf1r KD and control mice underwent laser speckle contrast imaging to assess neurovascular coupling at ∼12 months of age (Group 3) **(A-B)**. Representative pseudocolor laser speckle flowmetry maps of CBF (upper rows) and difference images reflecting CBF changes in the whisker barrel field relative to baseline during contralateral whisker stimulation (bottom rows) in control and IGF-1 receptor knockdown mice. Left and right images show representative images taken during the period before and after whisker stimulation. Center images show representative images taken during the whisker stimulation. Black and white circles reflect regions of interest (ROIs) placed over the somatosensory cortex. Arrows highlight the stimulated region. **(C)** Shown are the representative traces of the changes in CBF following contralateral whisker stimulation (horizontal bars). Summary data are shown in **(D)** (*n* = 6–7 in each group), *P < 0.05 by unpaired *t*-test. Plotted are means ± SEM with data points representing individual animals. Each value represents the average change in blood flow in the stimulated area of the brain compared to the baseline value in that same region (averaged from six sets of whisker stimulation).

Neurovascular coupling (NVC), a mechanism ensuring sufficient blood supply to regions of the brain with heightened neuronal demand, plays a vital role in cognitive function and is decreased in circulating IGF-1 deficient mice (Toth et al., 2015a). We evaluated NVC in normotensive mice (separate cohort from those that underwent autoregulation measurements) with VSMC-specific Igf1r KD by performing laser speckle imaging to assess changes in CBF in the somatosensory cortex in response to whisker stimulation [Figures 3A–C, black/white circles highlight regions of interest (ROI) selected for analysis, arrows show stimulated region]. Igf1r KD animals had a modest but statistically significant reduction in percent change in CBF in response to whisker stimulation compared to age-matched controls (Figure 3D), consistent with reduced NVC responses.

### Igf1r-deficiency in VSMCs leads to deficits in motor coordination and spatial learning in one-year old mice

**FIGURE 4:**
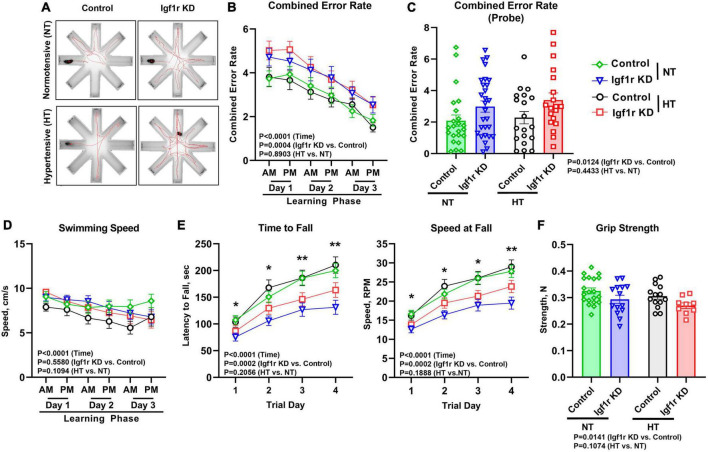
VSMC-specific Igf1r KD leads to impaired motor coordination and spatial learning. 4 weeks post-pump implantation, Group 2 mice began behavioral testing. Mice performed the radial arm water maze (RAWM) task. During the learning phase, mice performed this task four times per block with two blocks per day for 3 days. Seven days later, the mice were reintroduced to the maze and tested four times during the “probe” phase. **(A)** Shown are example traces (red lines) of the swimming path of a representative mouse from each group. **(B)** Plotted is combined error rate during the three-day learning phase and the **(C)** probe phase. The error rate is calculated based on the number of times a mouse enters an incorrect arm or is inactive in the center for 15 s. **(D)** Shown is the average swim speed of each group. **(E)** An accelerating rotating device (Rotarod) was used to assess motor coordination and learning in the mice. Mice performed the task three times per day for four consecutive days. Plotted is the time in seconds that mice fell off of the Rotarod (left), or the speed at fall (right). **(F)** Forelimb grip strength was measured in Newtons (N) using a grip strength meter with three sets of three trials per mouse. Data are shown as the mean ± SEM **(A–E)**. *n* = 16–26 mice pre group **(F)**. *n* = 9–20 mice/group **(B,D,E)**. Differences between groups were assessed using three-way ANOVA (variables time, normotensive [NT] vs. hypertensive [HT] and genotype) with Tukey’s *post-hoc* comparison, *p* values shown in each graph. *indicates pairwise comparisons between control normotensive and Igf1r KD normotensive (*P < 0.05, **P < 0.01). **(C, F)** Differences between groups were compared using two-way ANOVA with Tukey’s *post-hoc* correction. Plotted are mean ± SEM with data points representing individual animals.

Vascular cognitive impairment and dementia (VCID) is associated with cognitive and motor dysfunction. To assess hippocampal-dependent spatial learning and memory, Group 2 mice underwent evaluation using the radial arm water maze (RAWM, Figure 4A) beginning 4 weeks after pump implantation (AngII or saline). The combined error rate for the task is based on entries into the wrong arms and reflects performance; a lower combined error rate equates to better spatial learning and memory. During the three-day learning phase (two trial blocks per day) performance improved in all groups as animals learned the task; however, the Igf1r KD groups had significantly more errors over time than the control groups (*P* = 0.0004 for genotype by three-way ANOVA), a sign of impaired spatial learning (Figure 4B). 1 week after the learning phase, animals underwent the task again to assess memory (probe phase). Igf1r KD animals also performed significantly worse during this phase than the control animals during the probe phase (*P* = 0.0124 for genotype by two-way ANOVA), independent of whether they were hypertensive (Figure 4C). We also assessed short term and spatial working memory using the Y-maze. In the Y-maze, the time spent in familiar vs. unfamiliar maze arms was compared as a measure of working memory; however, no differences were observed between groups in Y-maze performance (Supplementary Figure 3).

To assess motor coordination and motor learning, we used an accelerating rotarod. Over 4 days, performance improved (as measured by time and speed at fall) as the mice learned the task. However, at all timepoints, Igf1r KD mice performed significantly worse than control mice, falling off sooner and at a lower speed (Figure 4E), independent of whether they were hypertensive. The differences grew day by day as the performance improved in the control groups (evidence of motor learning) with a lesser degree of improvement in the Igf1r KD group. In addition, our finding that speed at fall was lower in Igf1r KD compared to control at day one suggests that baseline motor coordination was also lower in Igf1r KD vs. control animals.

Because RAWM and rotarod measure cognitive performance using tasks that require physical activity, impaired strength could contribute to the observed defects in Igf1r KD mice. We therefore assessed swimming speed and grip strength (Figures 4D, F). While forelimb grip strength was slightly reduced in Igf1r KD animals vs. control animals, there were no differences in swimming speed between any groups, suggesting that overall physical strength does not underlie the defects we observed.

### Hypertension leads to microvascular rarefaction

**FIGURE 5:**
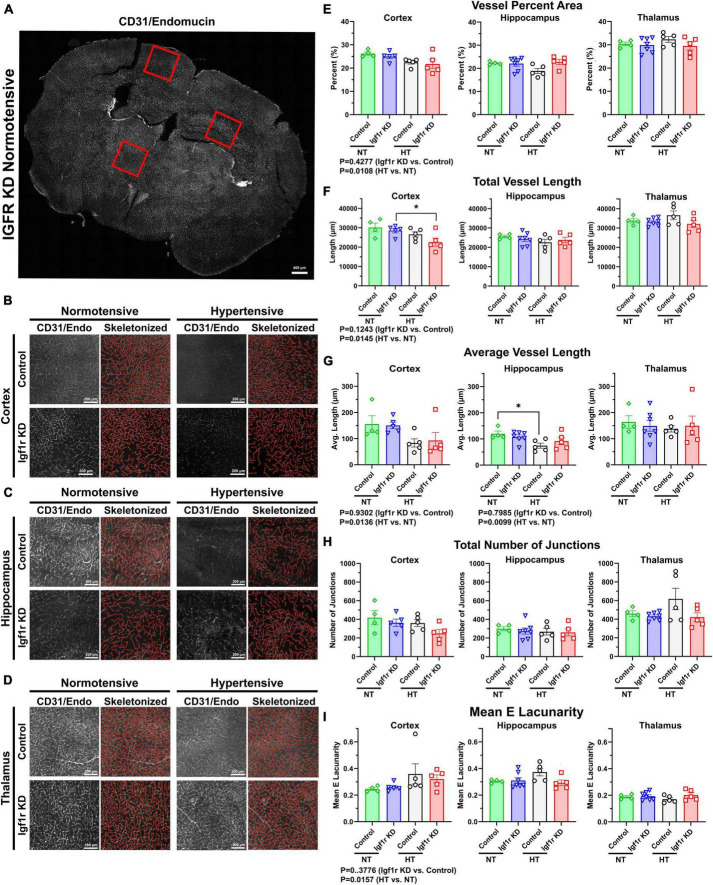
Hypertension leads to signs of microvascular rarefaction. **(A)** Example image of a 70 μm coronal section TileScan image labeled for endothelial cells (CD31and endomucin). Red squares highlight examples of areas (cortex/hippocampus/thalamus) where 2500 × 2500-pixel images were extracted for analysis **(B–D).** Example images extracted from cortex, hippocampus, and thalamus (left), with corresponding AngioTool skeletonization (right). **(E–I)** Quantification of vessel percent area, total vessel length, average vessel length, total number of junctions, and mean lacunarity was analyzed from Group 1 mice (4 weeks post pump-implantation). Each point represents the average value from multiple images from a single animal. Scalebar: 200 μm (*n* = 4–7 mice/group). Differences between groups were analyzed by two-way ANOVA [*p* values under graphs where either variable (genotype or hypertension) was significant] *P < 0.05 in Tukey’s *post-hoc* pairwise comparison. Plotted are mean ± SEM with data points representing individual animals.

**FIGURE 6:**
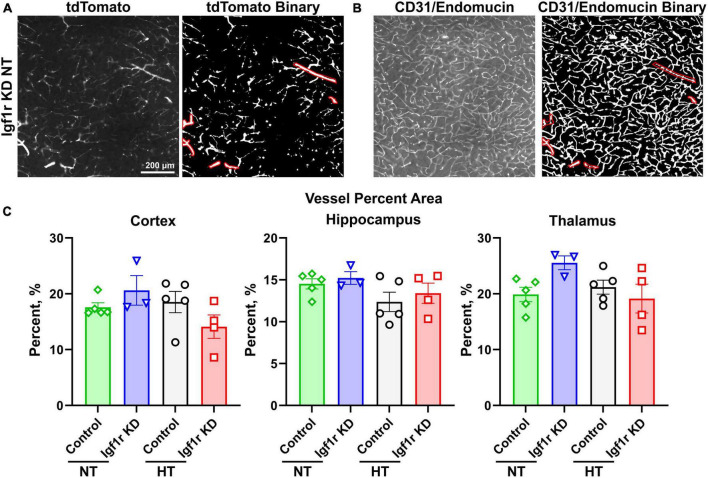
Analysis of capillary density. Coronal brain sections from normotensive/hypertensive (4 weeks post-pump implantation, Group 1) control and Igf1r KD mice were immunofluorescently labeled for CD31/endomucin and imaged for both native tdTomato fluorescence [left, **(A)**] and CD31/endomucin [left, **(B)**]. Binary images of both channels were created in ImageJ [right images in panel **(A,B)**], and ROIs corresponding to VSMC-covered vessels were created using the tdTomato binary image **(A)** based on the morphology of tdTomato positive cells. Those ROIs were applied to the CD31/endomucin binary image and removed from analysis. **(C)**. Plotted is vascular density of the region of the image not enclosed by ROIs (i.e., corresponding to small vessels). *n* = 3–5 mice/group, plotted is mean ± SEM. There were no significant differences between groups (two-way ANOVA).

Aged mice and mice with circulating IGF-1 deficiency show decreased capillary density in the brain (Tarantini et al., 2016). To assess this in VSMC-specific Igf1r KD animals, we performed immunofluorescent labeling for endothelial cells (using a cocktail of antibodies against CD31 and endomucin) on coronal brain sections cut through the hippocampus from Group 1 mice (4 weeks post-pump implantation, Figures 5, 6 and Supplementary Figure 4). Images were analyzed from specific brain regions (cortex, hippocampus, and thalamus, red squares Figure 5A). To evaluate multiple vascular parameters, images were analyzed using AngioTool (Zudaire et al., 2011). Representative images and their skeletonized counterparts are shown in Figures 5B–D. Using this method, we found that vessel percent area (a measure of vascular density) in the cortex was reduced in hypertensive vs. normotensive animals (*P* = 0.0108 by two-way ANOVA, Figure 5E), but genotype was not significant. A similar pattern was observed in other metrics: total vessel length and average vessel length were reduced and mean lacunarity (a measure of heterogeneity) increased in hypertensive vs. normotensive cortex (Figures 5E–I). Similarly, average vessel length was decreased in hypertensive vs. normotensive hippocampus (Figure 5G). To help validate these findings using an alternative approach, binary images were created in MATLAB from the same CD31/endomucin-labeled sections as were analyzed with AngioTool and vessel density was analyzed (Supplementary Figure 4). Vessel percent area in the hippocampus was reduced in hypertensive vs. normotensive animals, (*P* = 0.0088 by two-way ANOVA) and trended down in cortex but genotype (control vs. Igf1r KD) was not a significant contributor to variability. To see if genotype-based differences might become apparent at later timepoints, AngioTool-based measurements of vascular parameters were repeated on a Group 2 mice (7 weeks post-implantation), but no differences between control and Igf1r KD were observed (Supplementary Figure 5).

The forgoing analyses did not differentiate between capillaries and larger vessels. To separately analyze capillaries, we took advantage of the tdTomato reporter. Brain sections were stained for endothelial cells (CD31/endomucin) and imaged for both endothelial cells and tdTomato (Figures 6A, B, left image in each pair). Binary images were created for both channels (Figures 6A, B, right image in each pair), and ROIs were created using the tdTomato binary image in areas corresponding to VSMC-covered (red shows ROIs, Figure 6A) but not pericyte-covered vessels (determined based on morphology of the tdTomato positive cells). The ROIs corresponding to VSMC-covered vessels were then applied to the CD31/endomucin labeled binary image (red shows applied ROIs, Figure 6B) and excluded from analyses of vascular density. Analysis of non-VSMC-covered vessels [i.e., capillaries and pre-capillary arterioles (Berthiaume et al., 2018)] showed similar results to analyses of total vessels. We observed a trend toward decreased vascular density with hypertension, but the differences did not reach statistical significance and there were no differences between control and Igf1r KD animals (Figure 6C). Combined these data suggest that VSMC-specific Igf1r KD does not lead to microvascular rarefaction in the brain at ∼1 year of age.

### Igf1r KD leads to alterations in expression of genes associated with vascular dysfunction

**FIGURE 7:**
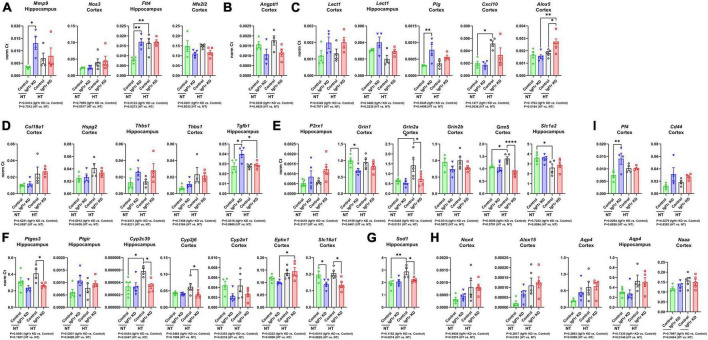
Igf1r KD induced changes in gene expression consistent with impaired vascular function. RNA was harvested from brain tissues (hippocampus or cortex) collected from Group 2 mice (7 weeks post -pump implantation). After cDNA synthesis, gene expression was analyzed for two panels of 96 genes associated with vascular structure and function (Supplementary Table 2). **(A–I)** Plotted are normalized Ct values for genes which were significantly changed. *n* = 4–5 samples/group. Differences between groups were analyzed by two-way ANOVA (*p*-values under each graph). *P < 0.05, **P < 0.01 by Tukey’s *post-hoc* pairwise comparison test. Plotted are means ± SEM with data points representing individual animals.

To help understand what molecular changes might be occurring in the brains of the VSMC-specific Igf1r KD animals, we evaluated genes associated with angiogenesis, rarefaction, and vascular function in cortex and hippocampus. We used qRT-PCR based assays (custom designed TaqMan microfluidics arrays) on tissues from Group 2 mice collected 7 weeks after mini-pump implantation (gene list in Supplementary Table 2). Genes that were significantly changed in these experiments are shown in Figure 7. We performed gene ontology (GO) analysis on the group of genes whose expression was altered (Supplementary spreadsheet 1, first tab), and since the genes in the panel were preselected to contain genes important for angiogenesis, neurovascular coupling, and vascular function, it was not surprising to find that among the GO terms that were significantly enriched with the list of changed genes were many associated with angiogenesis, cell migration, lipid metabolism, cell proliferation, reactive oxygen species, and cell signaling (including glutamate signaling).

To help further refine the potential roles played by the altered genes, we performed GO analysis on individual genes (Supplementary spreadsheet 1, beginning at tab 3). Each gene mapped to many different GO terms (significant terms are listed in the spreadsheet), but those that mapped to terms associated with angiogenesis are shown in Figures 7A–D. However, consistent with the lack of microvascular rarefaction in Igf1r KD brains, we did not observe changes in gene expression indicating an overall activation or inhibition of angiogenesis. We observed a few changes in genes that mapped to GO terms characteristic of a pro-angiogenic/anti-rarefaction phenotype [e.g., “sprouting angiogenesis” (GO:0002040), “positive regulation of angiogenesis” (GO:0045766, and “positive regulation of vasculature development” (GO:1904018)] in Igf1r KD brains such as upregulation of the VEGF receptor *Flt4* and *Mmp9*, and downregulation of the angiogenesis inhibitor *Angptl1* (Figures 7A, B) in Igf1r KD vs. control (Cao et al., 2006; Liu et al., 2018; Zhang et al., 2022; Thorin et al., 2023). However, we did not observe changes in other pro-angiogenic genes in the panel, including the growth factors *Vegfa, Vegfb, Vegfc, Figf, Pdgf*, or *Ctgf*. In addition, we observed upregulation of several other genes that mapped to anti-angiogenic GO terms such as “negative regulation of angiogenesis” (GO:0016525) genes including *Lect1, Plg, Alox5*, and *Cxcl10* (Shukunami and Hiraki, 2001; Lawler, 2002; Matsumoto et al., 2002; Bikfalvi, 2004; Yoshioka et al., 2006) in Igf1r KD vs. control (Figure 7C). We also observed changes in a few genes that mapped to GO terms associated with angiogenesis in general but either did not map specifically to terms associated with positive/negative regulation or mapped to both positive and negative regulation (Figure 7D). There was some variability between brain regions; for example, in the hippocampus, *Thbs1* was upregulated based on genotype (Igf1r KD vs. control) while in the cortex it was upregulated based on blood pressure (HT vs. NT, Figure 7D).

VSMCs in the vasculature respond to nitric oxide (NO) as part of multiple pathways, including NVC. We found signs that this pathway was modestly impaired in Igf1r KD brains vs. controls; *Thbs1* (an inhibitor of NO signaling that promotes vasoconstriction, Figure 7D) and *P2rx1* (a purinergic receptor whose activation promotes vasoconstriction) were upregulated in Igf1r KD brains compared to controls (Figure 7E). We also observed decreased expression of glutamate receptor subtypes important for NVC in Igf1r KD vs. control brains (*Grin1, Grin2a, Grin2b, Grm5*, Figure 7E). The idea that vascular tone may be dysregulated in Igf1r KD vs. control is further supported by altered expression of prostaglandin and eicosanoid-associated genes in Igf1r KD brains (*Ptges, Ptgir*; Figure 7F). *Cyp2c39*, a cytochrome P450 enzyme involved in the synthesis of the epoxyeicosanoids (EET) that promotes vasodilation during NVC, was upregulated in control hypertensive brains (vs. control normotensive), although this response was not present in Igf1r KD brains (Fisslthaler et al., 1999; Nayeem et al., 2008). However, *Ephx1*, an enzyme that degrades EETs, was also upregulated in hypertensive brains (Figure 7F) (Zhang et al., 2007). Lactate also plays a role in NVC (Cauli et al., 2023) and *Slc16a1*, a lactate transporter in endothelial cells and astrocytes, was downregulated in Igf1r KD vs. control (Figure 7F). Cellular antioxidant responses may be impaired in Igf1r KD, as several genes mapping to GO terms associated with positive regulation of cellular antioxidant systems such as *Nfe2l2* (Nrf2), *Sod1* (Figure 7G), and *Grin1*, were decreased in Igf1r KD vs. control. A few genes were upregulated with hypertension (rather than genotype), including some that mapped to GO terms associated with positive regulation of the cytoskeleton (e.g., *Nox4* and *Alox15*, Figure 7H). Many of the genes upregulated in the Igf1r KD mapped to GO terms associated with positive regulation of cell migration including *Mmp9, CD44, Flt4, Plg, Pf4* (Figure 7I), *Tgfb1, and Alox5*. Several altered genes mapped to GO terms associated with ECM remodeling or cell-matrix adhesion, including *Mmp9, Plg, Thbs1, Tgfb1, Col18a1, Hspg2*, and *Cyp2J6*. Combined, these gene changes support a brain environment associated with vascular dysregulation in VSMC-specific Igf1r KD animals. However, one challenge associated with these experiments is the presence of multiple cell types in our brain lysates, so narrowing down which of these gene expression changes could arise directly from VSMCs will require future single-cell based experiments.

## Discussion

Our findings suggest that IGF-1 signaling in VSMCs is important to maintain vascular functions such as autoregulation and NVC. Furthermore, this cerebrovascular impairment contributed to cognitive consequences, which include impaired motor coordination, motor learning, and spatial learning. These combined findings highlight the importance of VSMCs in brain health, particularly in phenotypes associated with VCID.

One of the interesting findings from this work was that hypertension did not exacerbate the effects of Igf1r KD on autoregulation and cognition. Hypertension is a major risk factor for other age-related cerebrovascular pathologies such as cerebral microhemorrhages, (Tarantini et al., 2017c) so we hypothesized that the additional insult of hypertension would worsen the phenotypes in our Igf1r KD group. However, we observed that phenotypes were worse in Igf1r KD mice regardless of the presence of elevated blood pressure. In previous studies using circulating IGF-1 knockdown animals and a similar hypertension paradigm to ours, autoregulatory responses were not different in normotensive circulating IGF-1 knockdown mice vs. controls; defects between groups were only seen in hypertensive animals (Toth et al., 2014). Similarly, middle cerebral arteries from IGF-1-deficient hypertensive mice did not exhibit pressure-induced constriction or pressure-induced increases in myogenic tone to the same degree as control mice or normotensive IGF-1 deficient animals (Toth et al., 2014). This discrepancy, that circulating IGF-1 knockdown impairs autoregulation only in hypertensive animals while VSMC-specific Igf1r impairs autoregulation in both normotensive and hypertensive animals, suggests that both circulating and locally produced IGF-1 in the CNS are important factors maintaining physiological responses. This may be especially relevant for vascular responses that rely on IGF-1 signaling in cells not directly exposed to the circulating IGF (such as VSMCs and astrocytes) where locally produced IGF-1 is likely to be a key source. While it remains possible that an extended hypertensive regimen may give rise to further alterations and/or maladaptations, our previous observations and the existing literature emphasizes that modifications in neurovascular coupling (NVC) and autoregulation stand out as acutely responsive to shifts in the environment.

The differences we observed in autoregulatory responses between control and VSMC-specific Igf1r KD mice likely have anatomical underpinnings. VSMCs undergo adaptive remodeling in response to a variety of stimuli, including hypertension. We previously found circulating IGF-1-deficient animals had thinner vascular walls than controls, independently of hypertension. IGF-1 promotes protective hypertrophy in the medial smooth muscle cell layer and VSMC hypertrophy *in vitro* (Bickel et al., 2023b). Several studies have focused on the effects of aging and IGF-1 deficiency on adaptive vessel remodeling in response to hypertension, showing that adaptive hypertrophy and ECM remodeling in larger arteries and penetrating arterioles is impaired in aged and circulating IGF-1-deficient animals (Toth et al., 2013, 2014; Tarantini et al., 2017c; Fulop et al., 2019b). However, our functional data showing physiological defects in normotensive Igf1r KD animals suggest that IGF-1 may also be important for basal maintenance of vascular wall integrity, not just for adaptive responses to hypertension. This is consistent with previous findings showing middle cerebral artery stiffening in normotensive circulating IGF-1 KD mice compared to controls (Fulop et al., 2019b). IGF-1 also plays a role in VSMC hypertrophy and ECM remodeling in other contexts. For example, IGF-1 is largely protective in atherogenesis, and IGF-1-mediated regulation of VSMC phenotype promotes stabilization of atherosclerotic plaques (Shai et al., 2011; von der Thusen et al., 2011; Bickel et al., 2023a). This is partly through IGF-1-mediated protective expression of ECM components such as *Col3a1* which can be suppressed by the inflammatory plaque environment (von der Thusen et al., 2011). Upregulation of *Col3a1* and other ECM regulators in the vascular wall is a key part of the cerebrovascular adaptive response to hypertension (Fulop et al., 2019b). One of the remaining questions is whether defective IGF-1 signaling in cerebrovascular VSMCs in aging prevents the adoption of protective VSMC phenotypes in the context of age-related challenges, such as hypertension and inflammation. Our data and an extensive literature on IGF-1-induced phenotypic switching suggest this is a likely possibility (Bickel et al., 2023b), but future studies may focus more explicitly on the cellular and molecular characteristics of brain VSMCs in the VSMC-specific Igf1r KD model.

Cerebrovascular autoregulation is critical for protecting downstream microvessels from pressure-induced damage, so we hypothesized that impaired autoregulation in the Igf1r KD animals would lead to microvascular rarefaction. However, we did not find significant microvascular rarefaction associated with VSMC-specific Igf1r KD, suggesting that the rarefaction seen in prior studies of circulating IGF-1 deficiency (Tarantini et al., 2016) is likely due to deficient IGF-1 signaling in endothelial cells rather than resulting from physiological abnormalities in VSMCs. In addition to rarefaction, there are several other manifestations of cerebrovascular fragility that occur in aging, many of which have also been seen with IGF-1 deficiency. These include increased BBB permeability (and neuroinflammation), and increased development of cerebral microhemorrhages (Toth et al., 2014; Braun et al., 2019; Towner et al., 2019; Verheggen et al., 2020a,b; Nyul-Toth et al., 2021; Montagne et al., 2022). Our recent findings indicate that endothelial cell-specific knockdown of Igf1r leads to increased BBB permeability, and we have studies in progress to evaluate whether impaired IGF-1 signaling on VSMCs also contributes to increased BBB permeability and increased cerebral microhemorrhages (Gulej et al., 2023). Like other phenotypes such as NVC (Toth et al., 2015a; Tarantini et al., 2021b), optimal blood-brain barrier integrity likely requires IGF-1 signaling in multiple cell types (e.g., endothelial cells, astrocytes, pericytes, and VSMCs) either due to a role for those cells in maintaining the BBB or due to a role in protecting vessels from blood pressure variations by pressure-induced constriction.

Our behavioral assessments showed impaired cognitive performance in Igf1r KD, particularly in motor coordination and spatial learning, that were not affected by concurrent hypertension. These findings suggest that VSMCs and their ability to regulate blood supply are crucial for optimal cognitive performance. The precise mechanistic links between VSMC function and cognition are unclear, but they are likely tied to the consequences of altered cerebral blood flow resulting from altered autoregulatory capacity. In addition, other physiological changes (apart from impaired autoregulation) likely contribute to decreases in cognitive performance including impaired NVC. During NVC, the neurovascular unit (comprised of endothelial cells, astrocytes, and pericytes) initiates vasodilation to promote increased blood flow in response to neuronal activation (Kisler et al., 2017; Toth et al., 2017). The vasodilation during NVC is mediated by relaxation of both pericytes and VSMCs. Age-related increases in pulsatile blood flow in the brain, which can arise due to arterial stiffening, contribute to the development of age-related cerebrovascular pathologies (Avolio et al., 2018). Cerebral arteries play a role in dampening this pulsatile flow, but this function is also reduced during aging (Zarrinkoob et al., 2016). While the exact molecular mechanisms underlying this dampening activity are not understood, optimal VSMC function is likely a key component.

Additional evidence for the role of VSMCs in cognition and VCID comes from the study of two vascular dementias, AD and cerebral autosomal dominant arteriopathy with subcortical infarcts (CADASIL). Studies in AD patients have shown that impairments in blood flow regulation and vascular reactivity occur in early stages of clinical disease (Kisler et al., 2017). Patients with mild to moderate AD had impaired vascular reactivity and autoregulation in response to hypercapnia and sit-stand maneuvers (den Abeelen et al., 2014). The Rotterdam Study, a large cohort study involving brain imaging and cognitive assessments, showed that cerebral hypoperfusion preceded clinical dementia (Ruitenberg et al., 2005). CADASIL is a genetic disorder caused by a Notch3 receptor mutation, and results in VSMC loss. The decreased VSMC-coverage of cerebral arteries and arterioles in CADASIL exacerbates vascular fragility associated with the disease (Kalimo et al., 2002; Tikka et al., 2014; Di Donato et al., 2017; Rajani et al., 2019; Chakraborty et al., 2021). This disease is clinically characterized by early-onset strokes that cause progressive cognitive impairment and neurological symptoms, demonstrating the importance of VSMCs to cerebrovascular health and cognition.

One limitation of this study is that because our Cre driver affects VSMCs throughout the body, we cannot formally rule out contributions from the systemic vasculature to the phenotypes we observed. In addition, another limitation is that because the Myh11 promoter drives Cre expression in both VSMCs and a subset of pericytes, it is not possible to distinguish effects that are due to VSMCs on arterioles/arteries vs. those due to effects on pericytes on smaller vessels. Pericytes have a variety of critical functions in the brain, including the maintenance of the BBB, NVC, angiogenesis, and clearance of toxic waste products (Uemura et al., 2020; Berthiaume et al., 2022). Because of their key roles in the vasculature, pericyte dysfunction and loss has been linked to VCID and other related diseases such as cerebral small vessel disease and Alzheimer’s disease (Sagare et al., 2013; Montagne et al., 2018; Liu et al., 2019; Nikolakopoulou et al., 2019; Uemura et al., 2020). Future studies may further evaluate the role of IGF-1 across different subtypes of brain mural cells via the use of single-cell-omics technologies.

In conclusion, these targeted studies have demonstrated a clear role for VSMCs in the development of aging-related brain pathologies associated with IGF-1 deficiency. Over the past several decades it has become clear that in the aging brain IGF-1 plays a vasoprotective role, with established benefits in astrocytes and endothelial cells. Here, we expand on this body of literature by demonstrating a role for IGF-1 in brain mural cells. VSMC-specific knockdown of Igf1r mimics many aging-associated changes, including cognitive impairment and impaired vascular physiology, and highlights the idea that impaired VSMC function may contribute to age-related cerebrovascular decline and VCID.

## Data availability statement

The original contributions presented in this study are included in this article/Supplementary materials, further inquiries can be directed to the corresponding author.

## Ethics statement

The animal study was approved by the University of Oklahoma Health Sciences Center Institutional Animal Care and Use Committee (IACUC). The study was conducted in accordance with the local legislation and institutional requirements.

## Author contributions

LM: Conceptualization, Formal analysis, Investigation, Methodology, Visualization, Writing – original draft, Writing – review & editing. MB: Formal analysis, Investigation, Visualization, Writing – original draft, Writing – review & editing. ST: Conceptualization, Funding acquisition, Investigation, Methodology, Resources, Writing – review & editing. MR: Investigation, Writing – review & editing. ZM: Writing – review & editing, Investigation. MV: Formal analysis, Investigation, Visualization, Writing – review & editing. CH: Investigation, Formal analysis, Writing – review & editing. HV: Investigation, Writing – review & editing. DN: Formal analysis, Investigation, Software, Visualization, Writing – review & editing. TM: Investigation, Writing – review & editing. EB: Investigation, Writing – review & editing. JP: Investigation, Writing – review & editing. TK: Formal analysis, Software, Writing – review & editing. EH: Investigation, Resources, Writing – review & editing. AY: Investigation, Methodology, Resources, Writing – review & editing. SC: Conceptualization, Formal analysis, Funding acquisition, Investigation, Methodology, Project administration, Resources, Supervision, Visualization, Writing – original draft, Writing – review & editing.

## Footnotes

1
https://geneontology.org/
