# Supplementary material for: IGF1R deficiency in vascular smooth muscle cells impairs myogenic autoregulation and cognition in mice
Source: Front Aging Neurosci. 2024 Feb 15;16:1320808. doi: 10.3389/fnagi.2024.1320808 (PMC10902040; doi:10.3389/fnagi.2024.1320808)
Supplement: Supplementary file 2 [file Data_Sheet_1.PDF]

Miller et al., Supplementary Material

Supplementary Figure 1

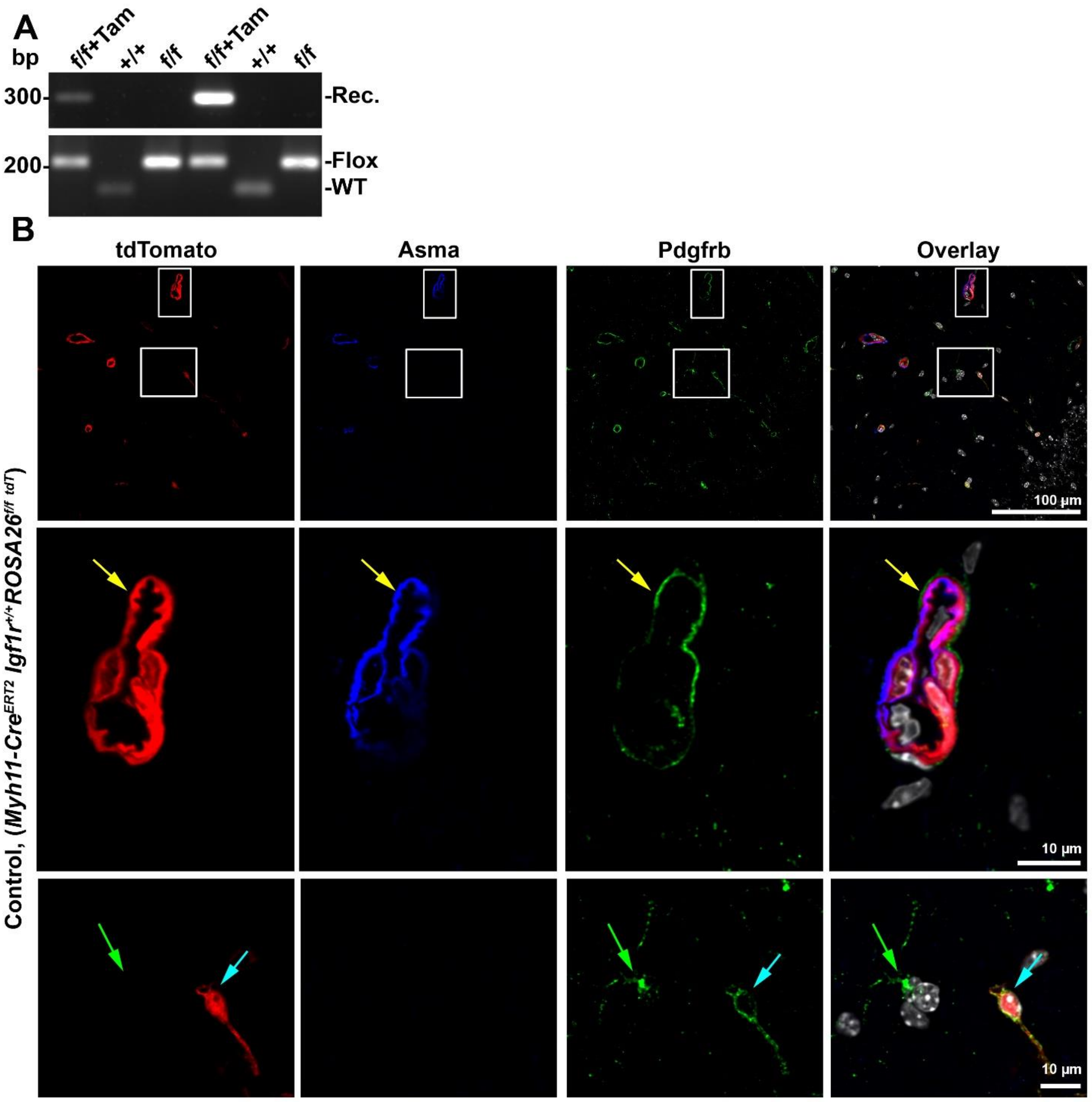

Supplementary Figure 1: Characterization of Myh11-Cre ERT2. A. Genotyping PCR of aortic samples (which contain a high number of VSMCs) was used to confirm the successful recombination of Lox P sites flanking exon 3 of the Igf1r gene in the *Myh11-Cre*^{ERT2} *Igf1r*$^{f/f}$ mouse line after tamoxifen injection. Top panel reflects amplicon only present in recombined Igfr flox allele. Bottom panel: larger amplicon reflects the floxed allele, bottom amplicon reflects the WT allele. **B.** Brain sections from *Myh11-Cre*^{ERT2}*Igf1r*$^{+/+}$*ROSA26*$^{f/f\ tdTomato}$ mice were co-labeled with antibodies for

VSMCs (Asma-alpha smooth muscle actin, blue), pericytes (Pdgfrb-platelet derived growth factor receptor β, green), and imaged along with endogenous tdTomato fluorescence (red) and nuclei (DAPI-grey). Shown are single confocal planes. Top row shows full field, middle row shows magnified version of top boxed region, bottom row shows magnification of bottom boxed region. Yellow arrows highlight VSMCs expressing tdTomato, blue arrows highlight pericytes expressing tdTomato, green arrows highlight pericytes not expressing tdTomato. Scale bars: 100 µm (top row), 10 µm (bottom row).

Supplementary Figure 2

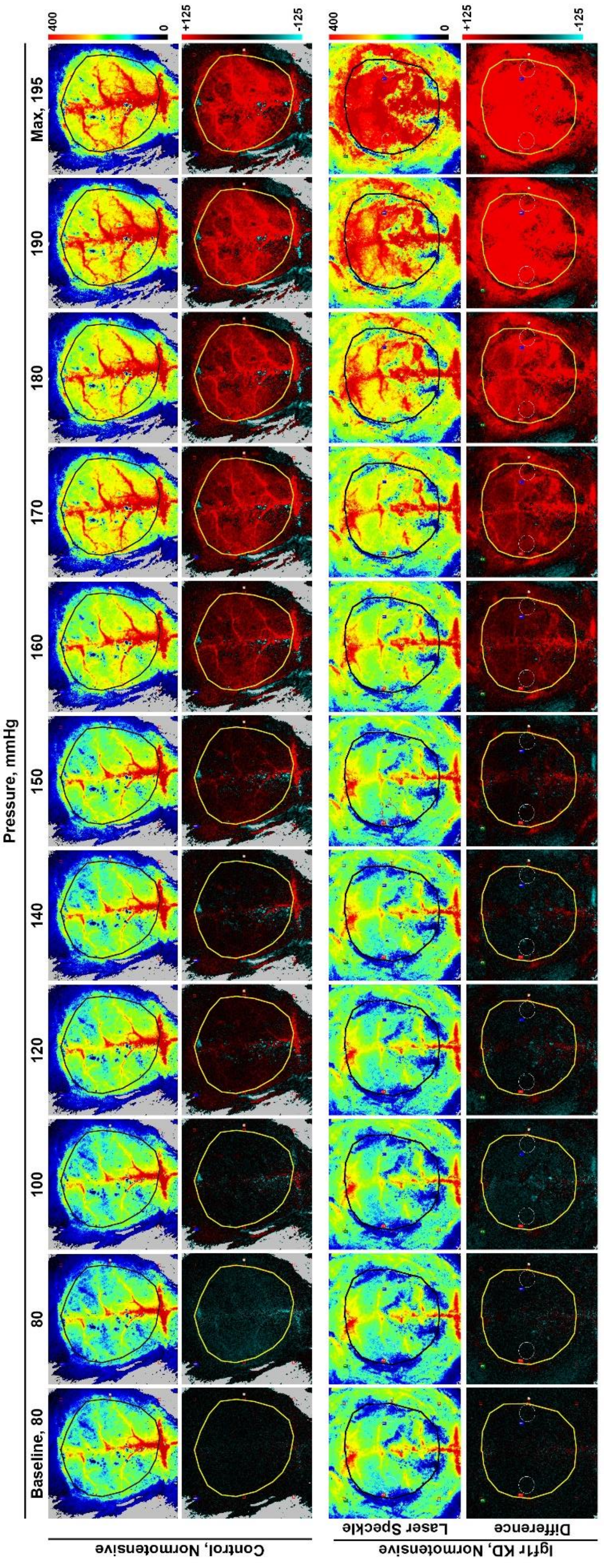

Supplementary Figure 2: VSMC-specific Igf1r KD impairs myogenic autoregulatory responses. To assess myogenic autoregulatory capacity in the VSMC-specific Igf1r KD model, we used laser speckle doppler imaging to measure changes in blood flow at increasing blood pressures on the cortical surface in mice with thin-skull cranial windows. Shown are example pseudocolor laser speckle images (top row of each set) and difference images (bottom row of each set) from each pressure step. In difference images, red indicates areas of blood flow that are elevated compared to baseline while blue indicates areas of blood flow reduced compared to baseline. Black/yellow outlines highlight the region used for analysis. Images come from the animals whose traces are depicted in **Fig. 2D** (top two rows) and **Fig. 2E** (bottom two rows). Baseline and 170 mmHg images are the same as in **Fig. 2B-C**.

Supplementary Figure 3

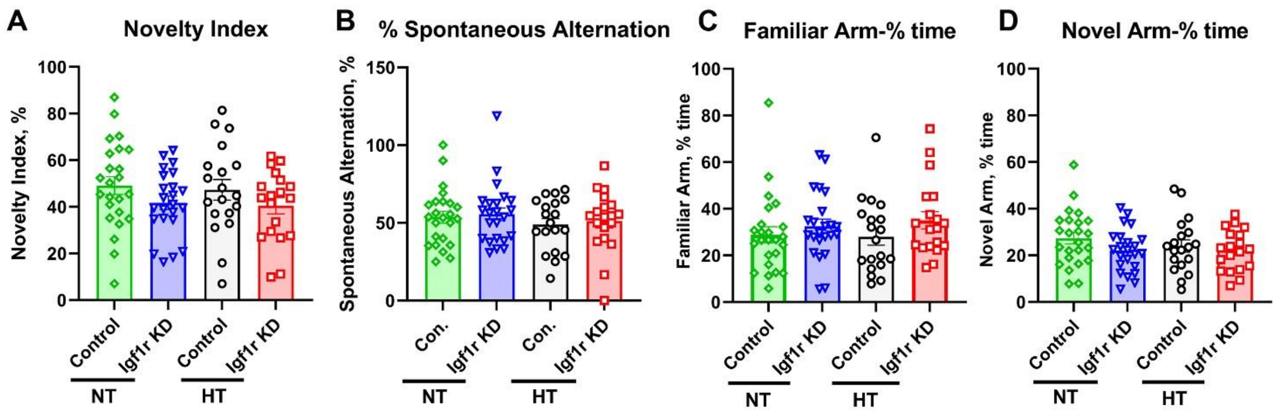

Supplementary Figure 3: Igf1r-deficiency in VSMCs does not affect Y-maze assay performance. To further assess hippocampal-dependent spatial memory, we performed the Y-maze assay following completion of the radial arm water maze assay (**Fig. 3**). Mice were acclimated to the Y-maze apparatus with only two arms available for exploration (the “home” and “familiar” arms). The mouse was then re-introduced to the apparatus four hours later with all three arms available to explore (the “novel” arm was made newly available). **A**. Plotted is novelty index, a reflection of spatial recognition of the unfamiliar part of the maze, spontaneous alternation (**B)** the inclination of the mice to explore new areas (arms) of the maze, and percent time spent in the familiar (**C**) or novel arm (**D**). n=19-24 mice/group. Differences between groups were assessed using two-way ANOVA with Tukey’s post-hoc comparison.

Supplementary Figure 4

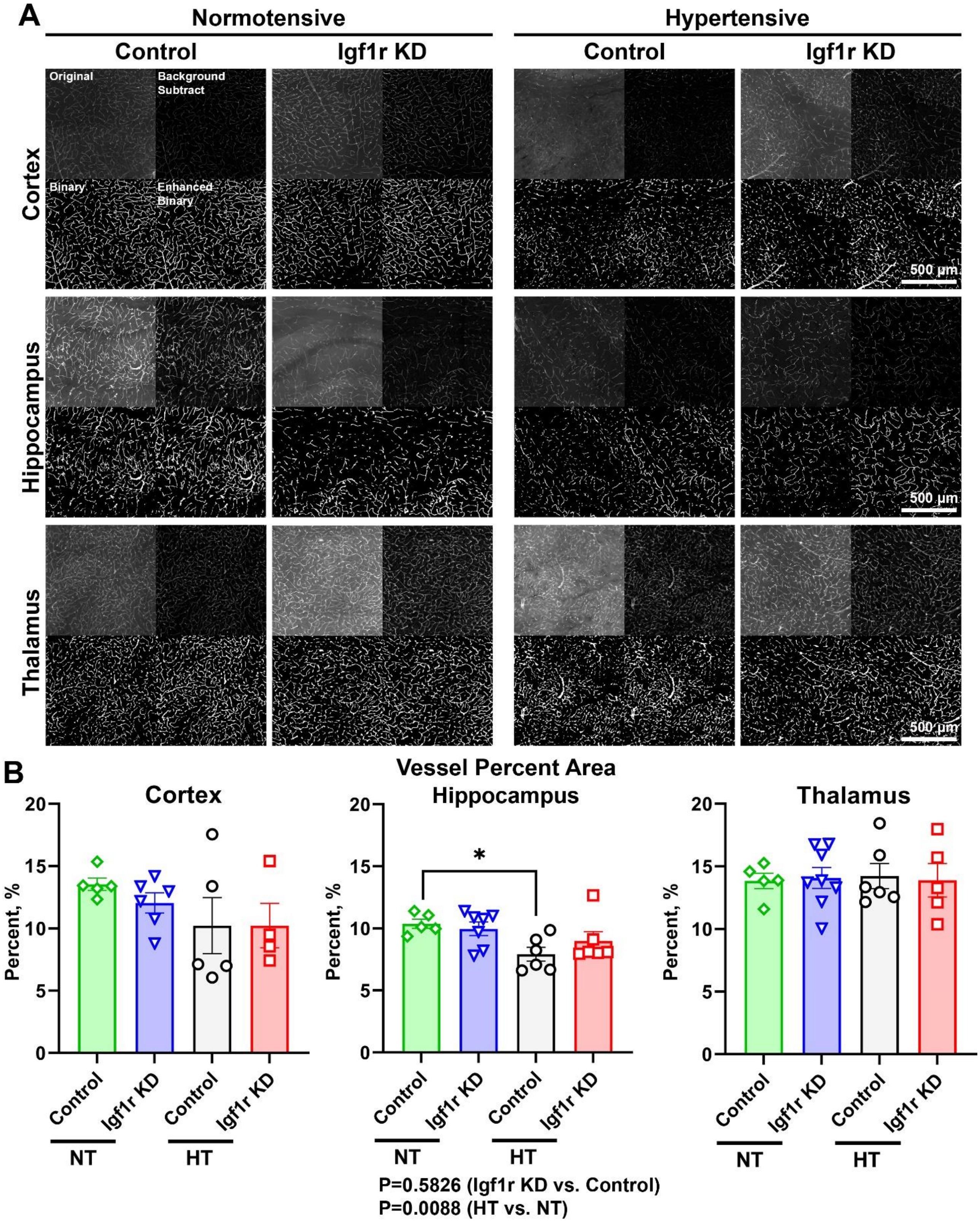

Supplementary Figure 4: Hypertension leads to microvascular rarefaction. Brains were sectioned, labeled for endothelial cells using antibodies against CD31 and endomucin, and imaged as in **Fig. 5.** Vascular density was analyzed using a MATLAB script. **A.** Each set of four tiled images shows the original image (top left), image after background subtraction (top right), simple binary (bottom left) and enhanced contrast binary (bottom right) image. **B.** Enhanced contrast binary images were used to analyze vessel percent area. Scalebar: 500 µm, n= 4-7 mice/group, each symbol reflects an average value from multiple images/mouse. Differences between groups were assessed by two-way ANOVA, p-values for graphs with significant differences are shown under each graph; *P<0.05 in Tukey's post-hoc pairwise comparison.

Supplementary Figure 5

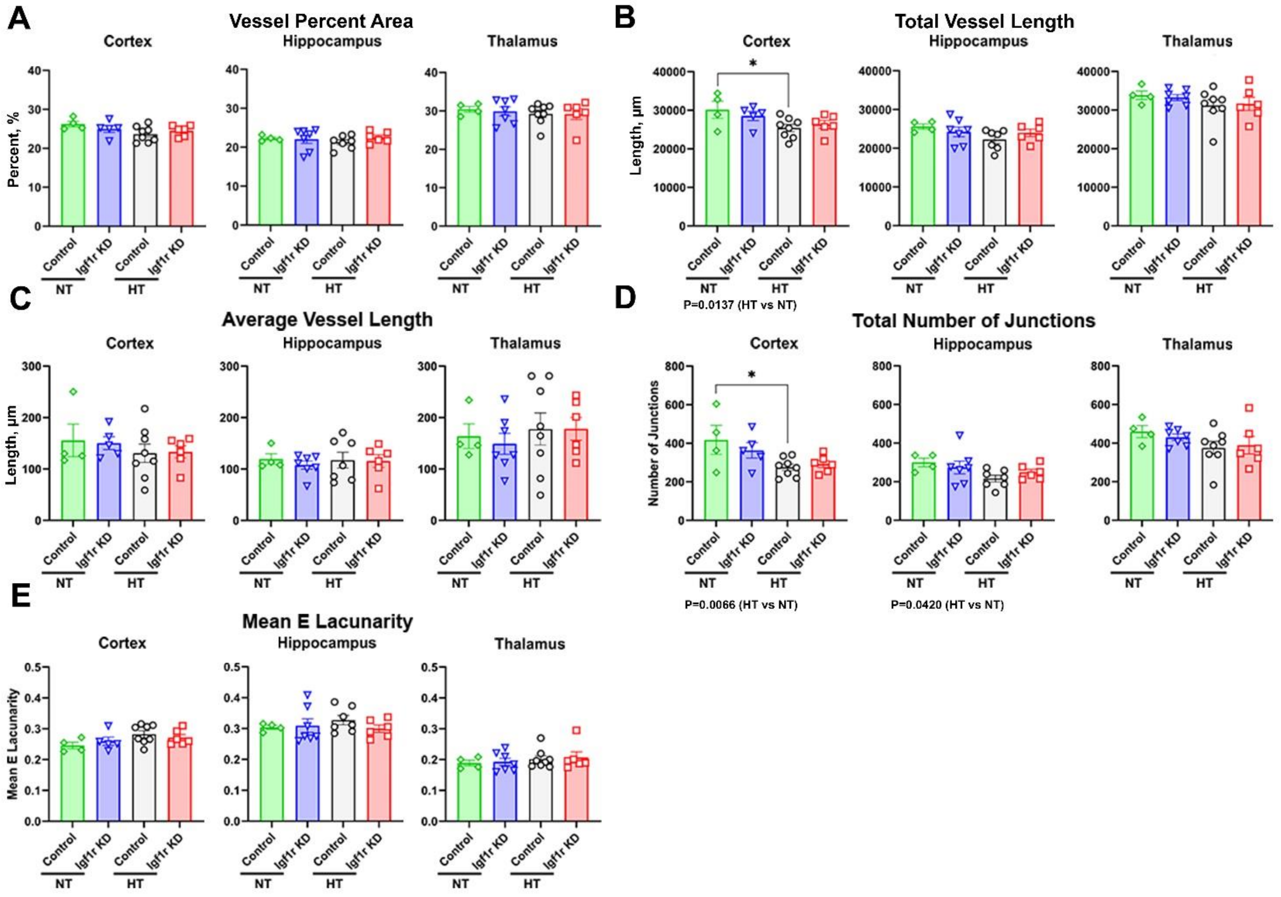

Supplementary Figure 5: Hypertension (7 weeks) leads to signs of microvascular rarefaction. A-E. Quantification of vessel percent area, total vessel length, average vessel length, total number of junctions, and mean lacunarity was performed using Group 2 brains and the Angiotool program. Each point represents the average value from multiple images from a single animal (n=4-7 mice/group). Differences between groups were analyzed by two-way ANOVA (p values under graphs where either variable [genotype or hypertension] was significant) *P<0.05 in Tukey's post-hoc pairwise comparison**.** Plotted are mean ±SEM with data points representing individual animals.

Supplementary Table 1

Gene	Primer Sequence	Primer Type
***Myh11-Cre**^{ERT2}*	TGA CCC CAT CTC TTC ACT CC	Transgene Forward
***Myh11-Cre**^{ERT2}*	AGT CCC TCA CAT CCT CAG GTT	Transgene Reverse
***Myh11-Cre**^{ERT2}*	CAG CCA ACT TTA CGC CTA GC	Internal Positive Control Forward
***Myh11-Cre**^{ERT2}*	TCT CAA GAT GGA CCT AAT ACG G	Internal Positive Control Reverse
Igf1r	CTT CCC AGC TTG CTA CTC TAG G	Igf1r KO and WT Forward
Igf1r	CAG GCT TGC AAT GAG ACA TGG G	Igf1r KO and WT Reverse
Igf1r	TGA GAC GTA GCG AGA TTG CTG TA	Recombination Forward
***ROSA** ^{tdT}*	AAG GGA GCT GCA GTG GAG TA	Wildtype Forward
***ROSA** ^{tdT}*	CCG AAA ATC TGT GGG AAG TC	Wildtype Reverse
***ROSA** ^{tdT}*	CTG TTC CTG TAC GGC ATG G	Mutant Forward
***ROSA** ^{tdT}*	GGC ATT AAA GCA GCG TAT CC	Mutant Reverse

Supplementary Table 1: List of genotyping primers. Above is a list of DNA primers used to genotype the mice during the experiments.

Supplementary Table 2

Angiogenesis Array			
**Gene Symbol(s)**	**Gene Name(s)**	**RefSeq(s)**	**Species**
*Ywhaz**	tyrosine 3-monooxygenase/tryptophan 5-monooxygenase activation protein, zeta polypeptide	NM_001253807.1;NM_001253806.1;NM_011740.3;NM_001253805.1	Mouse
*B2m**	beta-2 microglobulin	NM_009735.3	Mouse
*Hprt**	hypoxanthine guanine phosphoribosyl transferase	NM_013556.2	Mouse
Fga	fibrinogen alpha chain	-	Mouse
Plg	plasminogen	NM_008877.3	Mouse
Serpinc1	serine (or cysteine) peptidase inhibitor, clade C (antithrombin), member 1	NM_080844.4	Mouse
Prl	prolactin	NM_011164.2;NM_001163530.1	Mouse
Mmp2	matrix metallopeptidase 2	NM_008610.2	Mouse
Ang	angiogenin, ribonuclease, RNase A family, 5	NM_007447.3;NM_001161731.2	Mouse

Angpt1	angiopoietin 1	NM_009640.4;NM_001286062.1	Mouse
18s rRNA	-	-	Human
Angpt2	angiopoietin 2	NM_007426.4	Mouse
Cxcl12	chemokine (C-X-C motif) ligand 12	NM_001012477.2;NM_013655.4;NM_021704.3	Mouse
Edil3	EGF-like repeats and discoidin I-like domains 3	NM_001037987.3;NM_010103.4	Mouse
Ephb2	Eph receptor B2	NM_001290753.1;NM_010142.3	Mouse
Fgf1	fibroblast growth factor 1	NM_010197.3	Mouse
Fgf2	fibroblast growth factor 2	NM_008006.2	Mouse
Fgf4	fibroblast growth factor 4	NM_010202.5	Mouse
Fst	follistatin	NM_008046.3;NM_001301373.1;NM_001301375.1	Mouse
Hgf	hepatocyte growth factor	NM_001289459.1;NM_001289458.1;NM_001289460.1;NM_010427.5	Mouse
Cxcl15	chemokine (C-X-C motif) ligand 15	NM_011339.2	Mouse
Lep	leptin	NM_008493.3	Mouse
Mdk	midkine	NM_001291483.1;NM_010784.5;NM_001291481.1;NM_001012335.2;NM_001012336.2	Mouse
Tymp	thymidine phosphorylase	NM_138302.1	Mouse
Pdgfb	platelet derived growth factor, B polypeptide	NM_011057.3	Mouse
Ptn	pleiotrophin	NM_008973.2	Mouse
Prok1	prokineticin 1	NM_001044382.1	Mouse
Tgfa	transforming growth factor alpha	NM_031199.3	Mouse
Tgfb1	transforming growth factor, beta 1	NM_011577.1	Mouse
Tnf	tumor necrosis factor	NM_013693.3	Mouse
Vegfa	vascular endothelial growth factor A	NM_001110267.1;NM_001025250.3;NM_001110266.1;NM_001287057.1;NM_009505.4;NM_001287056.1	Mouse
Vegfb	vascular endothelial growth factor B	NM_001185164.1;NM_011697.3	Mouse
Vegfc	vascular endothelial growth factor C	NM_009506.2	Mouse
Ctgf	connective tissue growth factor	NM_010217.2	Mouse
Fbln5	fibulin 5	NM_011812.4	Mouse
Thbs1	thrombospondin 1	NM_011580.3	Mouse

Tnfsf15	tumor necrosis factor (ligand) superfamily, member 15	NM_177371.3	Mouse
Itga4	integrin alpha 4	NM_010576.3	Mouse
Ifnb1	interferon beta 1, fibroblast	NM_010510.1	Mouse
Ifng	interferon gamma	NM_008337.3	Mouse
Cxcl10	chemokine (C-X-C motif) ligand 10	NM_021274.2	Mouse
Il12a	interleukin 12a	-	Mouse
Serpinf1	serine (or cysteine) peptidase inhibitor, clade F, member 1	NM_011340.3	Mouse
Pf4	platelet factor 4	NM_019932.4	Mouse
Vash1	vasohibin 1	NM_177354.4	Mouse
Adamts1	a disintegrin-like and metallopeptidase (reprolysin type) with thrombospondin type 1 motif, 1	NM_009621.4	Mouse
Angptl1	angiopoietin-like 1	NM_028333.2	Mouse
Amot	angiomotin	NM_001290274.1;NM_153319.3	Mouse
Cd44	CD44 antigen	NM_009851.2;NM_001177787.1;NM_001039150.1;NM_001039151.1;NM_001177785.1;NM_001177786.1	Mouse
Cdh5	cadherin 5	NM_009868.4	Mouse
Cxcl2	chemokine (C-X-C motif) ligand 2	NM_009140.2	Mouse
Serpinb5	serine (or cysteine) peptidase inhibitor, clade B, member 5	NM_009257.3	Mouse
Flt1	FMS-like tyrosine kinase 1	NM_010228.3	Mouse
Sema3f	sema domain, immunoglobulin domain (Ig), short basic domain, secreted, (semaphorin) 3F	NM_011349.3	Mouse
Tek	endothelial-specific receptor tyrosine kinase	NM_001290551.1;NM_001290549.1;NM_013690.3	Mouse
Tie1	tyrosine kinase with immunoglobulin-like and EGF-like domains 1	NM_011587.2	Mouse
Tnmd	tenomodulin	NM_022322.2	Mouse
Timp2	tissue inhibitor of metalloproteinase 2	NM_011594.3	Mouse
Timp3	tissue inhibitor of metalloproteinase 3	NM_011595.2	Mouse
Angptl2	angiopoietin-like 2	NM_011923.4	Mouse
Angptl3	angiopoietin-like 3	NM_013913.3	Mouse
Ceacam1	carcinoembryonic antigen-related cell adhesion molecule 1	NM_001039187.1;NM_011926.2;NM_001039186.1;NM_001039185.1	Mouse

Hey1	hairy/enhancer-of-split related with YRPW motif 1	NM_010423.2	Mouse
Itgav	integrin alpha V	NM_008402.3	Mouse
Pecam1	platelet/endothelial cell adhesion molecule 1	-	Mouse
Lyve1	lymphatic vessel endothelial hyaluronan receptor 1	NM_053247.4	Mouse
Tnni1	troponin I, skeletal, slow 1	NM_021467.5;NM_001112702.1	Mouse
Nrp2	neuropilin 2	NM_001077406.1;NM_010939.2;NM_001077403.1;NM_001077407.1;NM_001077404.1;NM_001077405.1	Mouse
Kdr	kinase insert domain protein receptor	NM_010612.2	Mouse
Enpp2	ectonucleotide pyrophosphatase/phosphodiesterase 2	NM_001285995.1;NM_015744.3;NM_001285994.1;NM_001136077.2	Mouse
Figf	c-fos induced growth factor	-	Mouse
Foxc2	forkhead box C2	NM_013519.2	Mouse
Col4a1	collagen, type IV, alpha 1	NM_009931.2	Mouse
Col4a2	collagen, type IV, alpha 2	NM_009932.3	Mouse
Col15a1	collagen, type XV, alpha 1	NM_009928.3	Mouse
Hspg2	perlecan (heparan sulfate proteoglycan 2)	NM_008305.3	Mouse
Col18a1	collagen, type XVIII, alpha 1	NM_009929.3;NM_001109991.1	Mouse
Fn1	fibronectin 1	NM_001276409.1;NM_010233.2;NM_001276410.1;NM_001276408.1;NM_001276413.1;NM_001276411.1;NM_001276412.1	Mouse
Col4a3	collagen, type IV, alpha 3	NM_007734.2	Mouse
Adgrb1	adhesion G protein-coupled receptor B1	NM_174991.3	Mouse
Chga	chromogranin A	NM_007693.1	Mouse
Angpt4	angiopoietin 4	NM_009641.1	Mouse
Csf3	colony stimulating factor 3 (granulocyte)	NM_009971.1	Mouse
Grn	granulin	NM_008175.4	Mouse
Thbs2	thrombospondin 2	NM_011581.3	Mouse
Lect1	leukocyte cell derived chemotaxin 1	NM_010701.2	Mouse
Angptl4	angiopoietin-like 4	NM_020581.2	Mouse
Itgb3	integrin beta 3	NM_016780.2	Mouse

Pdgfra	platelet derived growth factor receptor, alpha polypeptide	NM_011058.2;NM_001083316.1	Mouse
Pdgfrb	platelet derived growth factor receptor, beta polypeptide	NM_008809.2;NM_001146268.1	Mouse
Flt4	FMS-like tyrosine kinase 4	NM_008029.3	Mouse
Nrp1	neuropilin 1	NM_008737.2	Mouse
S1pr1	sphingosine-1-phosphate receptor 1	NM_007901.5	Mouse
Prox1	prospero homeobox 1	NM_008937.2	Mouse
Mmp9	matrix metallopeptidase 9	NM_013599.3	Mouse
Hif1a	hypoxia inducible factor 1, alpha subunit	NM_010431.2	Mouse
Neurovascular Coupling Array			
**Gene Symbol(s)**	**Gene Name(s)**	**RefSeq(s)**	**Species**
*Actb**	actin, beta	-	Mouse
*Ywhaz**	tyrosine 3-monooxygenase/tryptophan 5-monooxygenase activation protein, zeta polypeptide	NM_001253807.1;NM_001253806.1;NM_011740.3;NM_001253805.1	Mouse
Polr2a	polymerase (RNA) II (DNA directed) polypeptide A	NM_001291068.1	Mouse
*Hprt**	hypoxanthine guanine phosphoribosyl transferase	NM_013556.2	Mouse
Gapdh	glyceraldehyde-3-phosphate dehydrogenase	NM_008084.3;NM_001289726.1	Mouse
Hmbs	hydroxymethylbilane synthase	NM_001110251.1;NM_013551.2	Mouse
Slc6a12	solute carrier family 6 (neurotransmitter transporter, betaine/GABA), member 12	NM_133661.3	Mouse
Slc6a13	solute carrier family 6 (neurotransmitter transporter, GABA), member 13	NM_144512.2	Mouse
Slc6a11	solute carrier family 6 (neurotransmitter transporter, GABA), member 11	NM_172890.3	Mouse
Slc1a3	solute carrier family 1 (glial high affinity glutamate transporter), member 3	NM_148938.3	Mouse
18s rRNA	-	-	Human
Slc1a1	solute carrier family 1 (neuronal/epithelial high affinity glutamate transporter, system Xag), member 1	NM_009199.2	Mouse
Slc1a2	solute carrier family 1 (glial high affinity glutamate transporter), member 2	NM_001077515.2;NM_001077514.3;NM_011393.2	Mouse

Slc16a1	solute carrier family 16 (monocarboxylic acid transporters), member 1	NM_009196.4	Mouse
Nos3	nitric oxide synthase 3, endothelial cell	NM_008713.4	Mouse
Nos1	nitric oxide synthase 1, neuronal	-	Mouse
Ptgs2	prostaglandin-endoperoxide synthase 2	NM_011198.3	Mouse
Ptgs1	prostaglandin-endoperoxide synthase 1	NM_008969.4	Mouse
Ptgds	prostaglandin D2 synthase (brain)	NM_008963.2	Mouse
Ptges	prostaglandin E synthase	NM_022415.3	Mouse
Ptges2	prostaglandin E synthase 2	NM_133783.2	Mouse
Ptges3	prostaglandin E synthase 3 (cytosolic)	NM_019766.4	Mouse
Akr1c21	aldo-keto reductase family 1, member C21	NM_029901.2	Mouse
Slco2a1	solute carrier organic anion transporter family, member 2a1	NM_033314.3	Mouse
Tbxa2r	thromboxane A2 receptor	NM_001277265.1;NM_009325.4	Mouse
Ptger1	prostaglandin E receptor 1 (subtype EP1)	NM_013641.2	Mouse
Ptgir	prostaglandin I receptor (IP)	NM_008967.3	Mouse
Ptger4	prostaglandin E receptor 4 (subtype EP4)	NM_001136079.2;NM_008965.2	Mouse
Alox5ap	arachidonate 5-lipoxygenase activating protein	-	Mouse
Alox5	arachidonate 5-lipoxygenase	NM_009662.2	Mouse
Alox12	arachidonate 12-lipoxygenase	NM_007440.4	Mouse
Alox15	arachidonate 15-lipoxygenase	NM_009660.3	Mouse
Mgst3	microsomal glutathione S-transferase 3	NM_025569.1	Mouse
Mgst2	microsomal glutathione S-transferase 2	NM_174995.2	Mouse
Alox8	arachidonate 8-lipoxygenase	NM_009661.4	Mouse
Ephx2	epoxide hydrolase 2, cytoplasmic	NM_001271403.1;NM_001271402.1;NM_001271421.1;NM_007940.4	Mouse
Ephx4	epoxide hydrolase 4	NM_001001804.2	Mouse
Ephx1	epoxide hydrolase 1, microsomal	NM_010145.2	Mouse
Cnr1	cannabinoid receptor 1 (brain)	NM_007726.3	Mouse
Faah	fatty acid amide hydrolase	NM_010173.4	Mouse
Cnr2	cannabinoid receptor 2 (macrophage)	-	Mouse

Cyp2e1	cytochrome P450, family 2, subfamily e, polypeptide 1	NM_021282.2	Mouse
Cyp4a14	cytochrome P450, family 4, subfamily a, polypeptide 14	NM_007822.2	Mouse
Cyp4a12b	cytochrome P450, family 4, subfamily a, polypeptide 12B	NM_172306.2	Mouse
Cyp4a10	cytochrome P450, family 4, subfamily a, polypeptide 10	NM_010011.3	Mouse
Cyp2c55	cytochrome P450, family 2, subfamily c, polypeptide 55	NM_028089.3	Mouse
Cyp2c37	cytochrome P450, family 2. subfamily c, polypeptide 37	NM_010001.2	Mouse
Cyp2j6	cytochrome P450, family 2, subfamily j, polypeptide 6	NM_010008.4	Mouse
Cyp2c29	cytochrome P450, family 2, subfamily c, polypeptide 29	NM_007815.3	Mouse
Cyp2c38	cytochrome P450, family 2, subfamily c, polypeptide 38	NM_010002.3	Mouse
Cyp2c67	cytochrome P450, family 2, subfamily c, polypeptide 69;cytochrome P450, family 2, subfamily c, polypeptide 67;cytochrome P450, family 2, subfamily c, polypeptide 40	NM_010004.2;NM_001024719.2;NM_001104525.1	Mouse
Cyp2c50	cytochrome P450, family 2, subfamily c, polypeptide 50	NM_001167875.1;NM_134144.2	Mouse
Cyp2c39	cytochrome P450, family 2, subfamily c, polypeptide 39	NM_010003.2	Mouse
Cyp2c54	cytochrome P450, family 2, subfamily c, polypeptide 54	NM_206537.2	Mouse
Cyp4x1	cytochrome P450, family 4, subfamily x, polypeptide 1	NM_001003947.1	Mouse
Cyp1a2	cytochrome P450, family 1, subfamily a, polypeptide 2	NM_009993.3	Mouse
Grm1	glutamate receptor, metabotropic 1	NM_001114333.2;NM_016976.3	Mouse
Grm5	glutamate receptor, metabotropic 5	NM_001081414.2;NM_001143834.1	Mouse
Grm4	glutamate receptor, metabotropic 4	NM_001013385.2;NM_001291045.1	Mouse
Grm2	glutamate receptor, metabotropic 2	NM_001160353.1	Mouse
Grm3	glutamate receptor, metabotropic 3	NM_181850.2	Mouse
Grin1	glutamate receptor, ionotropic, NMDA1 (zeta 1)	NM_001177657.2;NM_008169.3;NM_001177656.2	Mouse
Grin2c	glutamate receptor, ionotropic, NMDA2C (epsilon 3)	NM_010350.2	Mouse

Grin2b	glutamate receptor, ionotropic, NMDA2B (epsilon 2)	NM_008171.3	Mouse
Grin2a	glutamate receptor, ionotropic, NMDA2A (epsilon 1)	NM_008170.2	Mouse
Grin2d	glutamate receptor, ionotropic, NMDA2D (epsilon 4)	NM_008172.2	Mouse
Grin3a	glutamate receptor ionotropic, NMDA3A	NM_001276355.1;NM_001033351.2	Mouse
Nox4	NADPH oxidase 4	NM_015760.5;NM_001285835.1;NM_001285833.1	Mouse
Nox1	NADPH oxidase 1	NM_172203.2	Mouse
Cybb	cytochrome b-245, beta polypeptide	NM_007807.5	Mouse
Gclc	glutamate-cysteine ligase, catalytic subunit	NM_010295.2	Mouse
Nfe2l2	nuclear factor, erythroid derived 2, like 2	NM_010902.3	Mouse
Sod2	superoxide dismutase 2, mitochondrial	NM_013671.3	Mouse
Hmox1	heme oxygenase 1	NM_010442.2	Mouse
Nqo1	NAD(P)H dehydrogenase, quinone 1	NM_008706.5	Mouse
Gpx4	glutathione peroxidase 4	NM_008162.3	Mouse
Gpx1	glutathione peroxidase 1	NM_008160.6	Mouse
Pdgfb	platelet derived growth factor, B polypeptide	NM_011057.3	Mouse
Pdgfrb	platelet derived growth factor receptor, beta polypeptide	NM_008809.2;NM_001146268.1	Mouse
Adcyap1	adenylate cyclase activating polypeptide 1	NM_009625.2	Mouse
P2ry1	purinergic receptor P2Y, G-protein coupled 1	NM_008772.5	Mouse
P2ry2	purinergic receptor P2Y, G-protein coupled 2	-	Mouse
Adora2a	adenosine A2a receptor	NM_009630.3	Mouse
Adora2b	adenosine A2b receptor	NM_007413.4	Mouse
P2rx4	purinergic receptor P2X, ligand-gated ion channel 4	NM_011026.2	Mouse
P2rx1	purinergic receptor P2X, ligand-gated ion channel, 1	NM_008771.3	Mouse
Igf1r	insulin-like growth factor I receptor	NM_010513.2	Mouse
Igf1	insulin-like growth factor 1	NM_001111276.1;NM_001111275.1;NM_010512.4;NM_184052.3;NM_001111274.1	Mouse

Mgll	monoglyceride lipase	NM_011844.4;NM_001166249.1;NM_001166250.1;NM_001166251.1	Mouse
Naaa	N-acylethanolamine acid amidase	NM_001163687.1;NM_025972.4	Mouse
Cat	catalase	NM_009804.2	Mouse
Sod1	superoxide dismutase 1, soluble	NM_011434.1	Mouse
Sod3	superoxide dismutase 3, extracellular	NM_011435.3	Mouse
Arg1	arginase, liver	NM_007482.3	Mouse
Arg2	arginase type II	NM_009705.3	Mouse
Aqp4	aquaporin 4	NM_009700.2	Mouse

Supplementary Table 2: Curated gene lists for angiogenesis and neurovascular coupling arrays. Detailed above are the selected genes for both the angiogenesis and neurovascular coupling arrays described in this manuscript. Asterisks (*) denote genes selected for normalization of data.
